# Comparative Neuropharmacological Effects of Antiseizure Drugs on Cultured Myenteric and Dorsal Root Ganglion Neurons

**DOI:** 10.3390/ph19091356

**Published:** 2026-08-27

**Authors:** Aleksandr Subbotin, Holger A. Volk, Sebastian Meller, Gemma Mazzuoli-Weber, Kristin Elfers

**Affiliations:** 1 Department of Small Animal Medicine and Surgery, University of Veterinary Medicine Hannover Foundation, 30559 Hannover, Germanysebastian.meller@tiho-hannover.de (S.M.); 2 Center for Systems Neuroscience (ZSN), 30559 Hannover, Germany; gemma.mazzuoli-weber@tiho-hannover.de; 3 Institute for Physiology and Cell Biology, University of Veterinary Medicine Hannover Foundation, 30559 Hannover, Germany

**Keywords:** enteric nervous system, dorsal root ganglion, gut–brain axis, idiopathic epilepsy, topiramate, phenobarbital, levetiracetam, imepitoin, potassium bromide

## Abstract

**Background/Objectives:** Antiseizure drugs (ASDs) are the primary therapeutic approach for epilepsy in small animals. Although ASDs are primarily used to modulate central neuronal excitability, they are commonly administered systemically, most often by the oral route, and may therefore influence neuronal populations outside the central nervous system. Nevertheless, their functional effects on peripheral neuronal populations, including enteric and dorsal root ganglion (DRG) neurons, remain incompletely characterized at a comparative pharmacological level. This study aimed to perform a comparative functional neuropharmacological profiling of commonly used ASDs in primary cultured myenteric and DRG neurons. **Methods:** Changes in neuronal activity were assessed in primary cultured guinea pig myenteric and DRG neurons using voltage-sensitive dye imaging with Di-8-ANEPPS following direct ASD application under standardized in vitro conditions. **Results:** ASDs exerted distinct drug- and neuron-type-specific effects on peripheral neuronal excitability. Topiramate induced the most pronounced reduction in neuronal excitability in myenteric neurons, whereas phenobarbital and levetiracetam produced only minor changes compared with buffer control. Potassium bromide induced mainly excitatory effects in both enteric and DRG neurons. Overall, most ASDs predominantly increased neuronal excitability in DRG neurons. **Conclusions:** These findings demonstrate distinct functional response profiles of ASDs in enteric and sensory neuronal populations. This comparative in vitro approach may provide a basis for future studies investigating peripheral neuronal drug effects and may help relate experimental pharmacological profiling to clinically relevant challenges associated with ASD treatment across different disorders.

## 1. Introduction

Epilepsy is the most common chronic neurological disorder in both humans and dogs and is characterized by recurrent seizures resulting from neuronal hyperexcitability [1,2]. Although the etiology of epilepsy remains incompletely understood, lifelong treatment with antiseizure drugs (ASDs) represents the primary therapeutic strategy [2]. Despite pharmacological intervention, a substantial proportion of patients continue to experience seizures and may develop neurobehavioral comorbidities [3]. Variability in treatment response underscores the need for a more detailed understanding of ASD mechanisms at the cellular and molecular levels.

A major shared mechanism of action of several ASDs is the modulation of central γ-aminobutyric acid (GABA)-mediated inhibitory transmission [4]. Beyond GABAergic modulation, ASDs act through diverse additional mechanisms, including effects on voltage-gated sodium (Na^+^) and calcium (Ca^2+^) channels, synaptic vesicle protein 2A, and glutamatergic receptors such as α-amino-3-hydroxy-5-methyl-4-isoxazolepropionic acid (AMPA) and kainate receptors [5]. However, the exact mechanisms of action of some ASDs remain incompletely understood [6]. In canine clinical use, ASDs have been associated with gastrointestinal, neurological, metabolic, and occasional cutaneous adverse effects, including sedation, ataxia, vomiting, altered appetite, polyphagia or weight gain, weight loss, and others [7,8]. However, whether these clinically observed adverse effects are associated with direct ASD-induced changes in peripheral neuronal excitability remains insufficiently characterized.

Peripheral neurons, including those of the enteric nervous system (ENS) and dorsal root ganglia (DRG), play essential roles in gastrointestinal function, sensory processing, and bidirectional gut–brain communication [9,10]. Given that most ASDs are administered orally and undergo gastrointestinal absorption before systemic distribution, the ENS represents a pharmacologically relevant peripheral neuronal system for investigating non-central ASD effects, particularly in relation to gastrointestinal function. Moreover, within the concept of the gut–brain axis, modulation of enteric neuronal excitability may also influence the central nervous system (CNS) activity through bidirectional neural pathways [10]. DRG neurons represent a distinct sensory neuronal population involved in peripheral sensory and interoceptive processing and differ from enteric neurons in their morphology, functional organization, and excitability profiles [11]. Differences in ion channel expression and synaptic organization between enteric and DRG neurons may result in distinct pharmacodynamic response profiles. However, despite extensive characterization of ASD actions in the CNS, their direct and comparative effects on excitability in distinct peripheral neuronal populations have not been systematically examined under standardized in vitro conditions.

In a previous study investigating fecal microbiota transplantation (FMT) as a potential therapeutic approach in canine idiopathic epilepsy (IE) [12], we demonstrated that fecal supernatants (FS) from dogs with IE induced activity in cultured guinea pig myenteric neurons, with response patterns differing from those induced by FS from healthy controls. By contrast, phenobarbital alone, applied at the concentration detected in FS, did not affect baseline neuronal excitability. Further investigation of microbiota-associated effects in larger cohorts of dogs treated with different ASDs requires a complementary systematic evaluation of the direct effects of these compounds on peripheral neurons.

In the present study, we aimed to perform a comparative functional neuropharmacological profiling of commonly used ASDs in primary cultured guinea pig myenteric and DRG neurons across multiple concentrations under standardized in vitro conditions. Guinea pig myenteric and DRG neurons were used as established peripheral neuronal in vitro models to assess direct drug-induced effects on enteric and sensory neuronal excitability. This model allows assessment of direct drug-induced effects but does not directly reproduce canine in vivo treatment conditions. We hypothesized that ASDs differentially modulate neuronal excitability in enteric and DRG neurons, reflecting drug-, concentration-, and cell-type-specific pharmacodynamic properties.

## 2. Results

**Figure 1:**
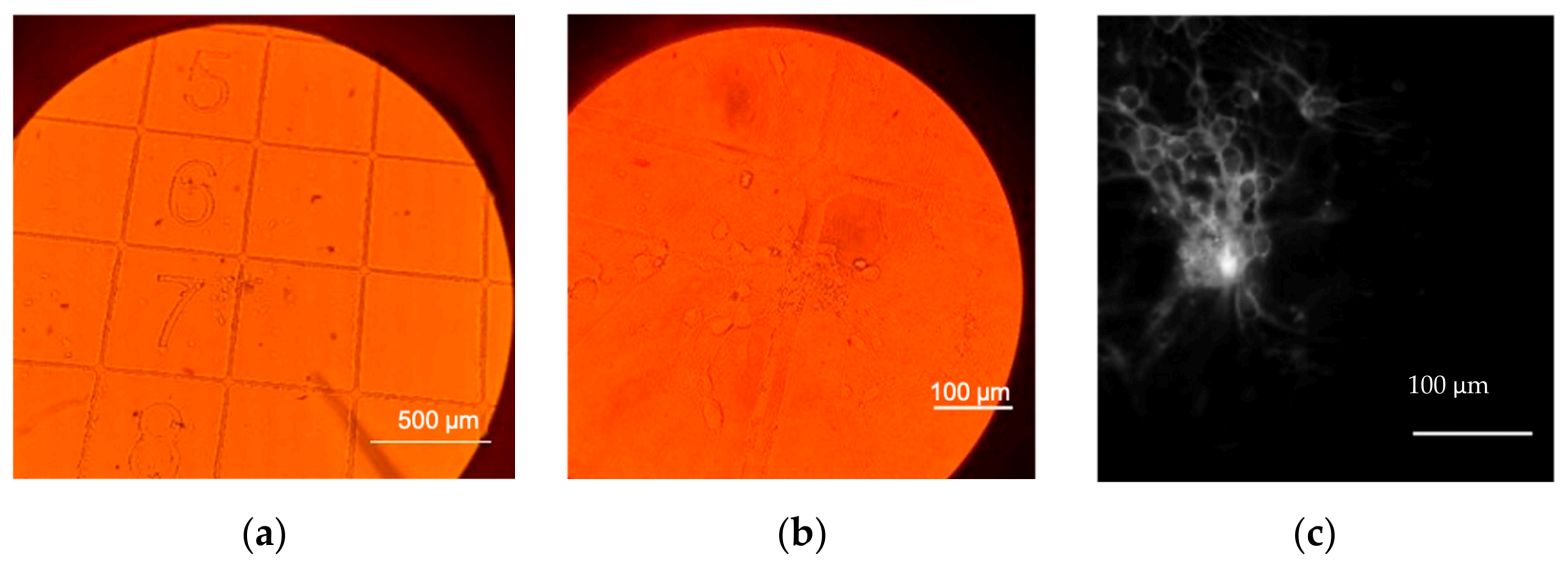
Representative images of an enteric neuronal cell cluster in a 12-day primary enteric neuronal culture prepared from guinea pig small intestine. (**a**) Bright-field image acquired with a 10× objective, showing part of one culture dish and the neuronal cell cluster at position 7. The shadow visible in the lower right corner is a glass micropipette positioned for local pressure application of the respective antiseizure drug. (**b**) Higher-magnification bright-field image of the same cluster acquired with a 40× objective. (**c**) Fluorescence image of the same cell cluster after staining with Di-8-ANEPPS, acquired using a 40× oil-immersion objective and CMOS camera, showing membrane labeling of the interconnected neuronal network. Scale bars: 500 µm in (**a**); 100 µm in (**b**,**c**).

**Figure 2:**
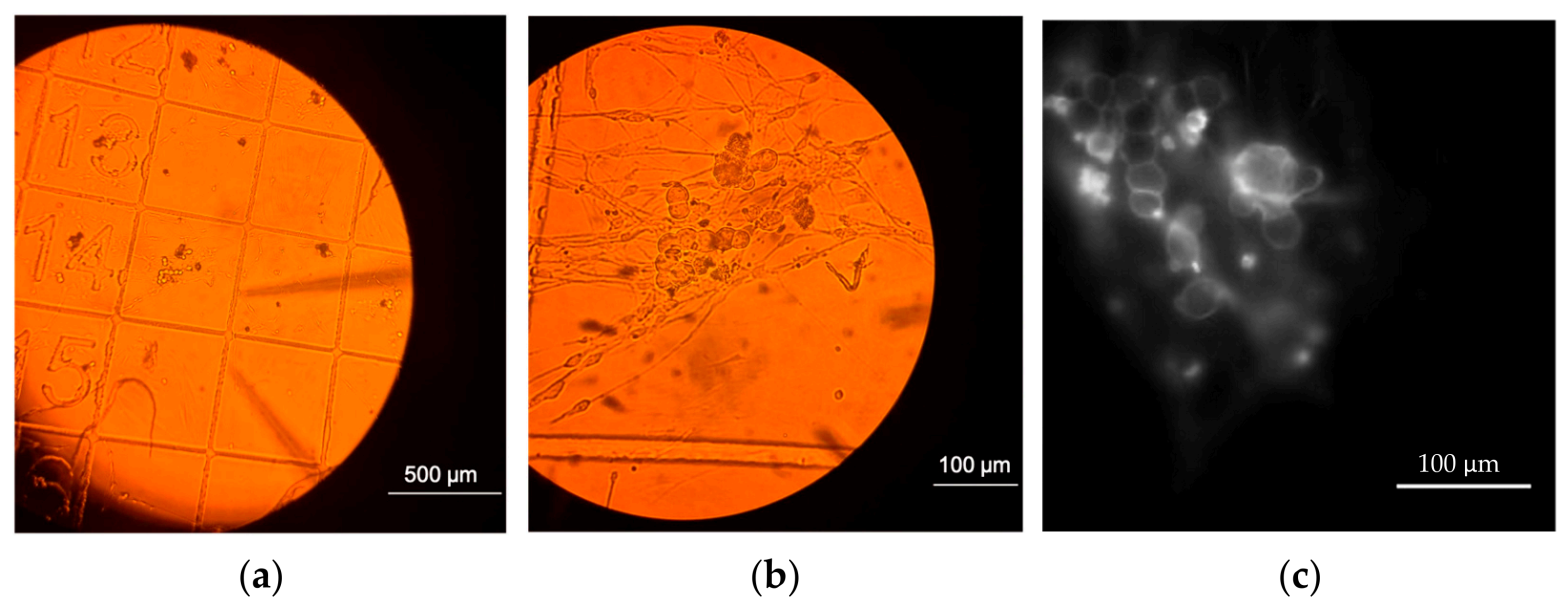
Representative images of a neuronal cluster in a 12-day primary dorsal root ganglion (DRG) neuronal culture prepared from a guinea pig. (**a**) Bright-field image acquired with a 10× objective, showing part of one culture dish and the neuronal cluster at position 14. The shadows visible in the lower right corner are glass micropipettes positioned for local pressure application of the respective antiseizure drug. (**b**) Higher-magnification bright-field image of the same cluster acquired with a 40× objective. (**c**) Fluorescence image of the same cluster after staining with Di-8-ANEPPS, acquired using a 40× oil-immersion objective and CMOS camera, showing membrane labeling of neuronal cell bodies and processes. Scale bars: 500 µm in (**a**); 100 µm in (**b**,**c**).

Representative images of cultured myenteric and DRG neurons are shown first to illustrate the neuronal cultures used for neuroimaging analysis (Figure 1 and Figure 2).

### 2.1. Phenobarbital

#### 2.1.1. Myenteric Neurons

The distribution of cluster response types following phenobarbital application was as follows: at a concentration of 40 µg/mL, phenobarbital induced no effect in 58.3% of clusters, an excitatory effect in 22.9%, an inhibitory effect in 14.6%, and a mixed effect in 4.2%. At 16.8 µg/mL, the distribution was 52.4% with no effect, 23.8% mixed, 11.9% excitatory, and 11.9% inhibitory. At 3.6 µg/mL, in 59.5% of clusters there was no effect, in 21.6% an inhibitory effect, in 13.5% an excitatory effect and in 8.1% a mixed effect (Figure S5a). Phenobarbital induced responses in a significantly greater proportion of neurons at 16.8 µg/mL compared to 40 µg/mL (*p* = 0.0474). No other significant differences were observed between phenobarbital concentrations or compared to the Krebs buffer solution, and phenobarbital did not induce significant changes in action potential (AP) number compared with baseline activity.

After Benjamini–Hochberg false discovery rate (FDR) procedure correction, none of the phenobarbital-induced effects in myenteric neurons remained significant.

#### 2.1.2. DRG Neurons

The distribution of cluster response types following phenobarbital application was as follows: at a concentration of 40 µg/mL, phenobarbital induced excitatory effects in 49.3% of clusters, no effect in 37.3%, a mixed effect in 10.4%, and an inhibitory effect in 3.0%.

At 16.8 µg/mL, the distribution was 53.1% excitatory, 36.7% with no effect, 8.2% inhibitory, and 2.0% mixed. At 3.6 µg/mL, in 56.1% of clusters an excitatory effect was recorded, in 29.3% no effect, in 12.2% a mixed effect, and in 2.4% an inhibitory effect (Figure S5b). Phenobarbital induced responses in a significantly greater proportion of neurons per cluster at 40 µg/mL compared to 3.6 µg/mL (*p* = 0.014) and Krebs buffer solution (*p* = 0.0125) (Figure S9a). The number of APs did not differ significantly between phenobarbital concentrations or compared to the Krebs buffer solution; however, in comparison with baseline activity, phenobarbital induced a significant increase in AP number at 40 µg/mL (*p* < 0.001), 16.8 µg/mL (*p* < 0.001), and 3.6 µg/mL (*p* < 0.001) (Table 1). Phenobarbital induced significantly higher burst frequencies at 40 µg/mL (*p* = 0.038) and 16.8 µg/mL (*p* = 0.047) compared to the Krebs buffer solution (Figure S9b). The duration of AP firing was significantly shorter at 16.8 µg/mL compared to the Krebs buffer solution (*p* = 0.0238) (Figure S9c). No other significant differences were observed between phenobarbital concentrations or compared to the Krebs buffer solution.

After FDR correction, the increase in AP number compared with baseline activity remained significant at all phenobarbital concentrations in DRG neurons.

### 2.2. Potassium Bromide (KBr) and Potassium Gluconate (KGlu)

Results for KBr and the intergroup comparison with KGlu are presented below. Additional detailed analyses for KGlu and further supporting figures are provided in the Supplementary Materials.

**Figure 3:**
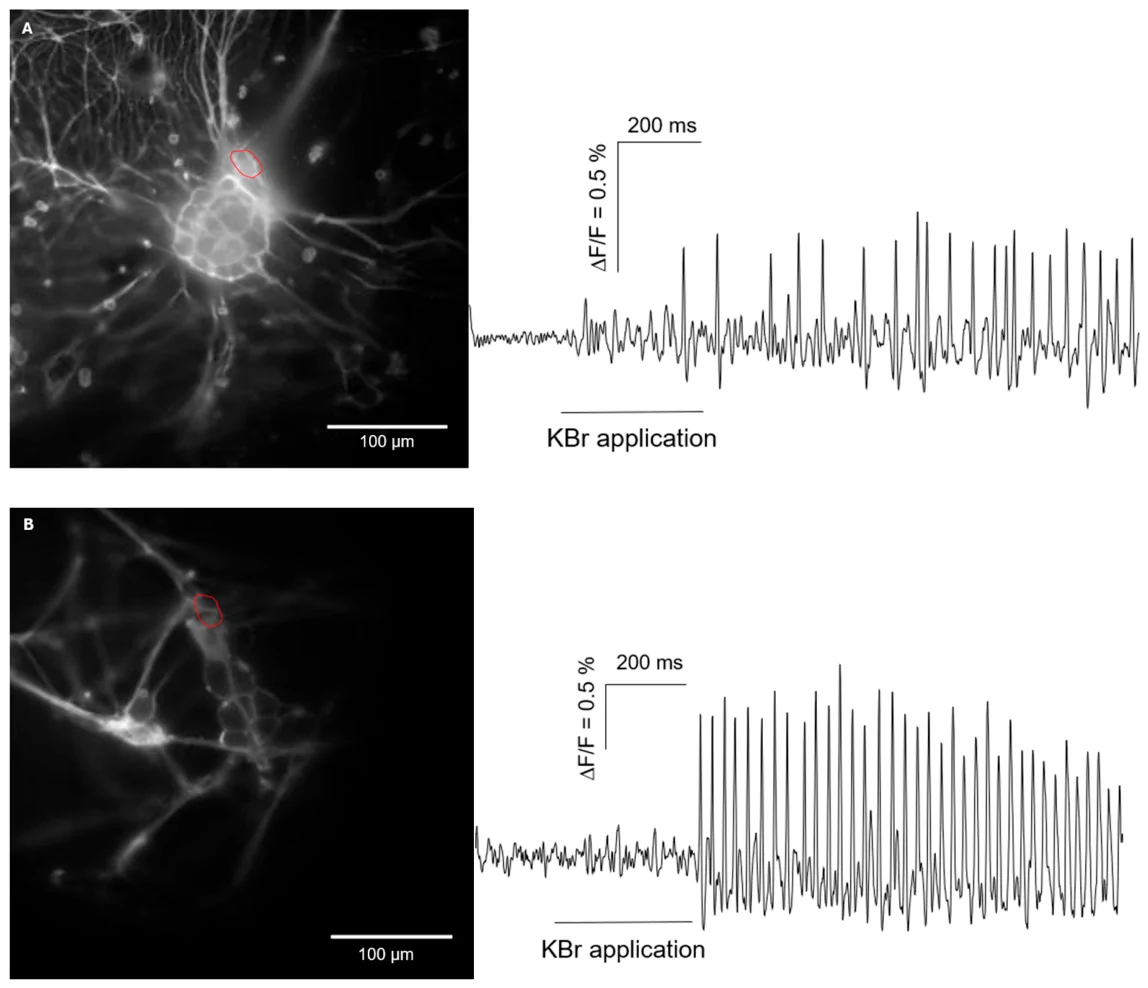
Action potential discharge in cultured guinea pig primary myenteric and dorsal root ganglion (DRG) neurons evoked by KBr application at 100 mg/mL. Representative Di-8-ANEPPS-loaded images show a primary myenteric neuronal cluster (**A**) and a DRG neuronal cluster (**B**) cultured for 12 days. The neurons selected for trace analysis are encircled in red. The corresponding optical recording traces are shown next to each image. KBr was applied for 500 ms, as indicated by the bar below each trace, and evoked action potential discharge in the marked neurons. Scale bars: 100 µm.

Representative Di-8-ANEPPS-loaded images and optical recording traces of myenteric and DRG neurons after KBr application are shown in Figure 3.

#### 2.2.1. Myenteric Neurons

**Figure 4:**
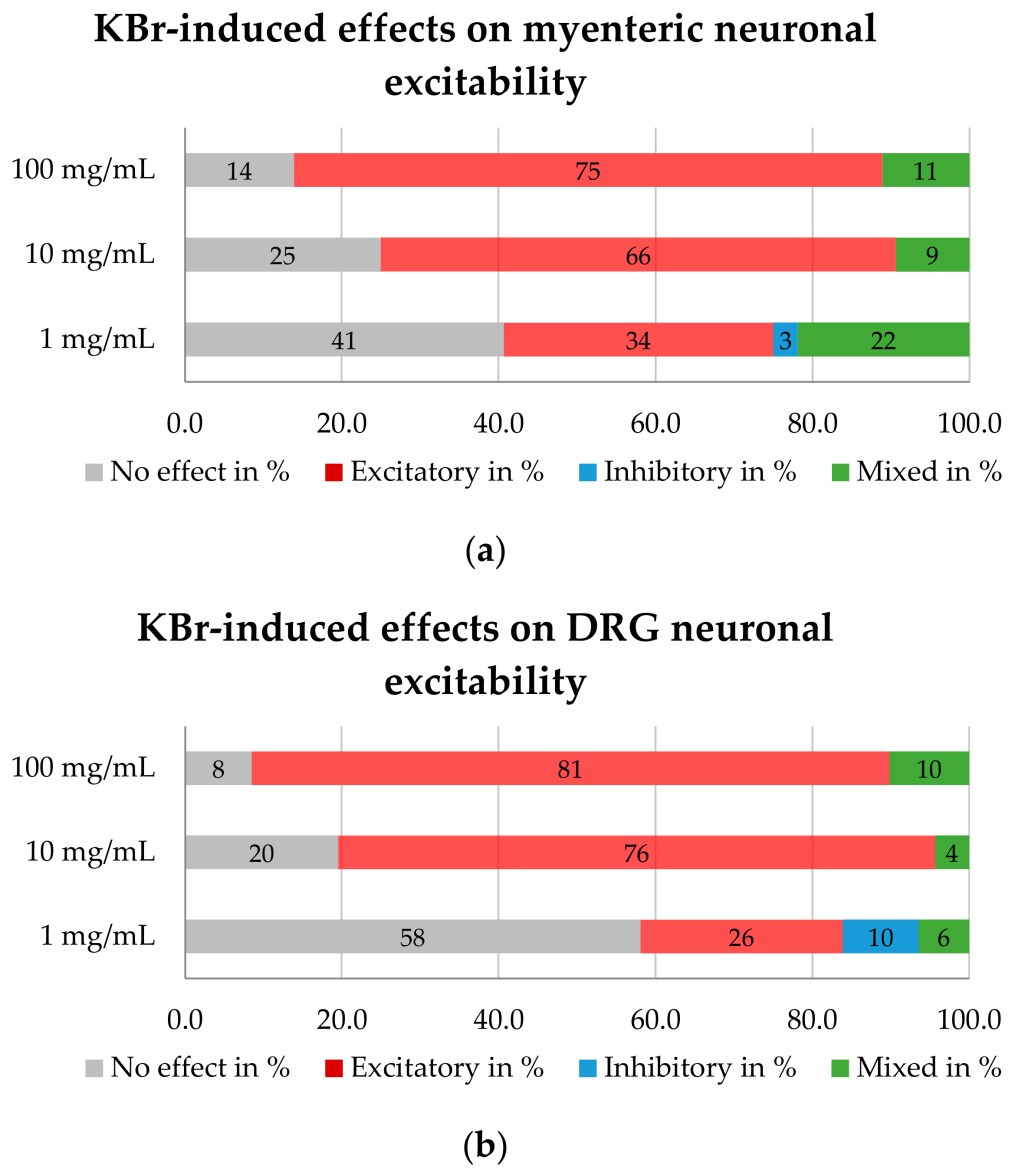
Potassium bromide (KBr)-induced effects on neuronal excitability at the cluster level in myenteric (**a**) and dorsal root ganglion (DRG) neuronal cultures (**b**). Stacked horizontal bar charts show the proportion of neuronal clusters in which application of KBr induced no effect (gray), excitatory (red), inhibitory (blue), or mixed excitatory/inhibitory effects (green) on neuronal excitability at concentrations of 100, 10, and 1 mg/mL.

The distribution of cluster response types following KBr application was as follows: at a concentration of 100 mg/mL, KBr induced excitatory effects on neuronal excitability in 75.0% of clusters, no effect in 13.9%, and mixed effects in 11.1%. At 10 mg/mL, the distribution was 65.6% excitatory, 25.0% with no effect, and 9.4% mixed. At 1.0 mg/mL, in 40.6% of clusters there was no effect, in 34.4% an excitatory effect, in 21.9% a mixed effect, and in 3.1% an inhibitory effect (Figure 4a).

**Figure 5:**
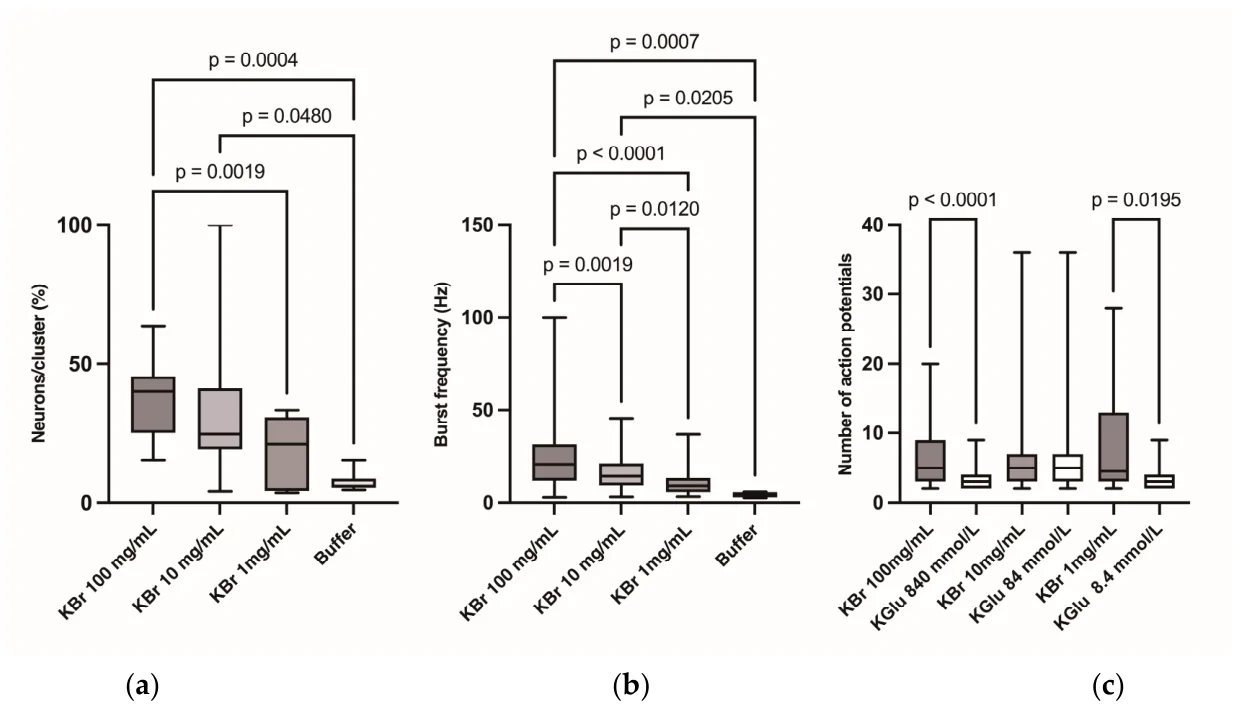
Effects of potassium bromide (KBr) on myenteric and dorsal root ganglion (DRG) neuronal excitability. Data are shown as box-and-whisker plots (median, interquartile range, minimum–maximum). Significant differences between conditions, including comparisons between concentrations and buffer, are indicated (Dunn’s multiple comparisons test; *p*-values shown). (**a**) Myenteric neurons: neurons per cluster (%). Percentage of myenteric neurons per cluster exhibiting drug-induced changes in activity following application of KBr at 100, 10, and 1 mg/mL and Krebs buffer solution. (**b**) DRG neurons: burst frequency (Hz). Burst frequency of DRG neurons following application of KBr at 100, 10, and 1 mg/mL and Krebs buffer solution. (**c**) Myenteric neurons: action potential (AP) number (KBr vs. potassium gluconate (KGlu)). Number of APs recorded in myenteric neurons following application of KBr (100, 10, and 1 mg/mL) and equimolar potassium gluconate (KGlu; 840, 84, and 8.4 mmol/L).

KBr induced responses in a significantly greater proportion of neurons per cluster at 100 mg/mL compared with 1 mg/mL (*p* = 0.0019) and Krebs buffer solution (*p* = 0.0004), and at 10 mg/mL compared with Krebs buffer solution (*p* = 0.0480) (Figure 5a). No significant difference was observed between KBr and KGlu at equimolar potassium (K^+^) concentrations.

KBr induced a significantly higher number of APs at 100 mg/mL compared with Krebs buffer solution (*p* = 0.0277) (Figure S10a). Compared with baseline activity, KBr induced a significant increase in AP number at 100 mg/mL (*p* < 0.001), 10 mg/mL (*p* < 0.001), and 1 mg/mL (*p* = 0.0002) (Table 1). In direct equimolar comparisons, KBr at 100 mg/mL induced significantly more APs than KGlu at 840 mmol/L (*p* < 0.0001), and KBr at 1 mg/mL induced more APs than KGlu at 8.4 mmol/L (*p* = 0.0195) (Figure 5c).

KBr induced significantly higher burst frequencies at 100 mg/mL compared with Krebs buffer solution (*p* = 0.0115) and 1 mg/mL (*p* = 0.0452) (Figure S10b).

KBr induced a significantly longer duration of AP firing at 1 mg/mL compared with 100 mg/mL (*p* = 0.0130) (Figure S10c). In direct equimolar comparisons, KBr at 1 mg/mL induced a significantly longer duration of AP firing than KGlu at 8.4 mmol/L (*p* = 0.0303) (Figure S12a).

After FDR correction, the following KBr effects remained significant: responder proportion at 100 mg/mL vs. 1 mg/mL and Krebs buffer; AP number vs. baseline at all concentrations; AP number for 100 mg/mL KBr vs. 840 mmol/L KGlu; burst frequency at 100 mg/mL vs. Krebs buffer; and AP firing duration at 1 mg/mL vs. 100 mg/mL.

#### 2.2.2. DRG Neurons

The distribution of cluster response types following KBr application was as follows: at a concentration of 100 mg/mL, KBr induced excitatory effects in 81.4% of clusters, mixed effects in 10.2%, and no effect in 8.5%. At 10 mg/mL, the distribution was 76.1% excitatory, 19.6% with no effect, and 4.3% mixed. At 1.0 mg/mL, in 58.1% of clusters no effect was recorded, in 25.8% an excitatory effect, in 9.7% an inhibitory effect and in 6.5% a mixed effect (Figure 4b).

KBr induced responses in a significantly greater proportion of neurons per cluster at 100 mg/mL compared to 1 mg/mL (*p* = 0.0009) and Krebs buffer solution (*p* = 0.0029) (Figure S11a). No significant differences were detected between KBr and KGlu at equimolar K^+^ concentrations.

KBr induced a significantly higher number of APs at 100 mg/mL compared with 10 mg/mL (*p* = 0.0071) and 1 mg/mL (*p* < 0.0001), and at 10 mg/mL compared with 1 mg/mL (*p* = 0.0013) (Figure S11b). In comparison with baseline activity, KBr induced a significant increase in AP number at 100 mg/mL (*p* < 0.0001), 10 mg/mL (*p* < 0.0001), and 1 mg/mL (*p* = 0.0094) (Table 1). In direct equimolar comparisons, KBr induced a significantly higher number of APs than KGlu at 10 mg/mL vs 84 mmol/L (*p* < 0.0001) (Figure S12c).

KBr induced significantly higher burst frequencies at 100 mg/mL compared to 10 mg/mL (*p* = 0.0019), 1 mg/mL (*p* < 0.0001), and Krebs buffer solution (*p* = 0.0007). At 10 mg/mL, KBr also induced significantly higher burst frequencies compared to 1 mg/mL (*p* = 0.0120) and Krebs buffer solution (*p* = 0.0205) (Figure 5b).

KBr induced a significantly shorter duration of AP firing at 10 mg/mL (*p* = 0.0499) and 1 mg/mL (*p* = 0.0429) compared to Krebs buffer solution (Figure S11c). In direct equimolar comparison, KBr at 10 mg/mL induced a significantly longer duration of AP firing than KGlu at 84 mmol/L (*p* = 0.0078) (Figure S12b).

After FDR correction, the following KBr effects remained significant in DRG neurons: responder proportion at 100 mg/mL vs. 1 mg/mL and Krebs buffer; AP number at 100 mg/mL vs. 10 mg/mL and 1 mg/mL, and at 10 mg/mL vs. 1 mg/mL; AP number vs. baseline at all concentrations; AP number for 10 mg/mL KBr vs. 84 mmol/L KGlu; and burst frequency at 100 mg/mL vs. 10 mg/mL, 1 mg/mL, and Krebs buffer, and at 10 mg/mL vs. 1 mg/mL.

### 2.3. Imepitoin

#### 2.3.1. Myenteric Neurons

The distribution of cluster response types following imepitoin application was as follows: at a concentration of 100 µg/mL, imepitoin induced no effect in 92.6% of clusters, an excitatory effect in 3.7%, and an inhibitory effect in 3.7%. At 50 µg/mL, the distribution was 78.6% with no effect, 10.7% inhibitory, 7.1% mixed, and 3.6% excitatory. At 10 µg/mL, in 46.9% of clusters there was no response to Imepitoin, in 22.4% an inhibitory effect was recorded, in 20.4% a mixed effect, and in 10.2% an excitatory effect (Figure S6a). No significant differences in the percentage of responding neurons, AP number, burst frequencies, or duration of AP firing were recorded for the investigated imepitoin concentrations compared to the 1% dimethyl sulfoxide (DMSO) control. In comparison with baseline activity, imepitoin did not induce significant changes in AP number at any concentration.

#### 2.3.2. DRG Neurons

The distribution of cluster response types following Imepitoin application was as follows: at a concentration of 100 µg/mL, imepitoin induced no effect in 80.6% of clusters, an inhibitory effect in 16.7%, and an excitatory effect in 2.8%. At 50 µg/mL, the distribution was 77.5% with no effect, 12.5% excitatory, 7.5% inhibitory, and 2.5% mixed. At 10 µg/mL, in 66.7% of clusters there was no effect, in 25.6% an excitatory effect, in 5.1% an inhibitory effect, and in 2.6% a mixed effect (Figure S6b). No significant differences in the percentage of responding neurons, AP number, burst frequencies, or duration of AP firing were recorded after application of imepitoin at 10 or 50 µg/mL compared to the 1% DMSO control. Parameters could not be calculated at 100 µg/mL because no APs were recorded. In comparison with baseline activity, AP number was reduced after imepitoin application at 10 µg/mL (*p* = 0.0207), and complete suppression was observed at 100 µg/mL (*p* = 0.0045), whereas at 50 µg/mL no significant differences were recorded compared to baseline (Table 1).

The 1% DMSO control itself induced a significant increase in AP number compared to baseline activity (*p* = 0.0019) (Table 1).

After FDR correction, the AP suppression observed at 100 µg/mL imepitoin remained significant in DRG neurons; the 10 µg/mL comparison did not remain significant, whereas the DMSO-induced increase in AP number also remained significant.

### 2.4. Levetiracetam

#### 2.4.1. Myenteric Neurons

The distribution of cluster response types following levetiracetam application was as follows: at a concentration of 100 µg/mL, levetiracetam induced no effect in 66.7% of clusters, a mixed effect in 16.7%, an inhibitory effect in 11.1%, and an excitatory effect in 5.6%. At 50 µg/mL, the distribution was 51.5% with no effect, 21.2% excitatory, 18.2% inhibitory, and 9.1% mixed. At 10 µg/mL, in 58.8% of clusters there was no effect, in 23.5% an inhibitory effect, in 11.8% an excitatory effect, and in 5.9% a mixed effect (Figure S7a). Compared to Krebs buffer solution, no significant differences in the percentage of responding neurons or in AP numbers were recorded after levetiracetam application. In comparison with baseline activity, levetiracetam induced a significant reduction in AP number at 50 µg/mL (*p* = 0.0025) and 10 µg/mL (*p* = 0.0002) (Table 1). Burst frequencies and the duration of AP firing did not differ significantly between levetiracetam concentrations or compared to the Krebs buffer solution.

After FDR correction, the reduction in AP number compared with baseline activity at 50 µg/mL and 10 µg/mL levetiracetam remained significant in myenteric neurons.

#### 2.4.2. DRG Neurons

The distribution of cluster response types following levetiracetam application was as follows: at a concentration of 100 µg/mL, levetiracetam induced excitatory effects in 43.8% of clusters, no effect in 41.7%, a mixed effect in 12.5%, and an inhibitory effect in 2.1% of tested clusters. At 50 µg/mL, the distribution was 39.6% excitatory, 39.6% with no effect, 14.6% inhibitory, and 6.3% mixed. At 10 µg/mL, in 52.1% of clusters there was no effect, in 25.0% an excitatory effect, in 14.6% an inhibitory effect, and in 8.3% a mixed effect (Figure S7b). Levetiracetam at 50 µg/mL induced responses in a significantly greater proportion of neurons per cluster compared with 100 µg/mL (*p* = 0.0315), 10 µg/mL (*p* = 0.0029), and Krebs buffer solution (*p* = 0.0115) (Figure S13a). In comparison with baseline activity, levetiracetam induced a significant increase in AP number at 100 µg/mL (*p* < 0.001) and 50 µg/mL (*p* < 0.001) (Table 1). Burst frequency was significantly higher at 100 µg/mL compared to the Krebs buffer solution (*p* = 0.0215) (Figure S13b). No other significant differences were observed between levetiracetam concentrations and the Krebs buffer solution.

After FDR correction, only the increase in AP number compared with baseline activity at 100 µg/mL and 50 µg/mL levetiracetam remained significant in DRG neurons.

### 2.5. Topiramate

#### 2.5.1. Myenteric Neurons

**Figure 6:**
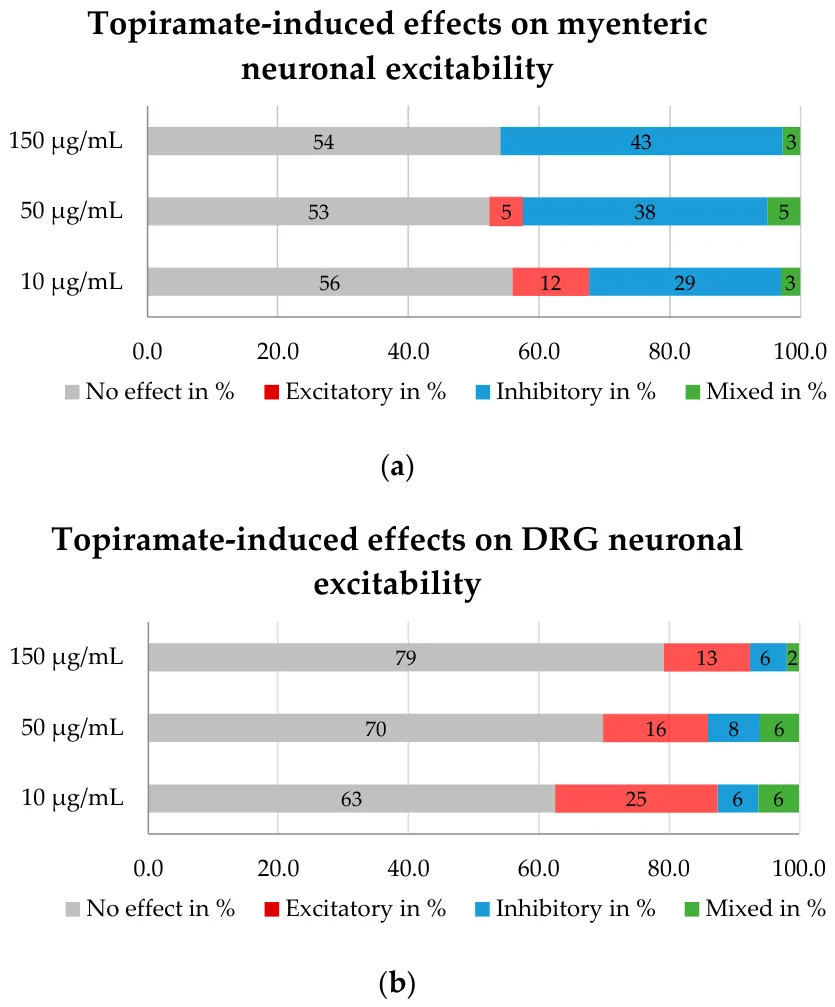
Topiramate-induced effects on neuronal excitability at the cluster level in myenteric (**a**) and dorsal root ganglion (DRG) neuronal cultures (**b**). Stacked horizontal bar charts show the proportion of neuronal clusters in which application of topiramate induced no effect (gray), excitatory (red), inhibitory (blue), or mixed excitatory/inhibitory effects (green) on neuronal excitability at concentrations of 150, 50, and 10 µg/mL.

The distribution of cluster response types following topiramate application was as follows: at a concentration of 150 µg/mL, topiramate induced no effect in 54.1% of clusters, an inhibitory effect in 43.2%, and a mixed effect in 2.7%. At 50 µg/mL, the distribution was 52.5% with no effect, 37.5% inhibitory, 5.0% excitatory, and 5.0% mixed. At 10 µg/mL, in 55.9% of clusters there was no effect, in 29.4% an inhibitory effect, in 11.8% an excitatory effect, and in 2.9% a mixed effect (Figure 6a). No significant differences were recorded in the percentage of responding neurons among the investigated concentrations of topiramate or compared to the Krebs buffer solution. In comparison with baseline activity, topiramate induced a significant reduction in AP number at 150 µg/mL (*p* < 0.0001), 50 µg/mL (*p* = 0.0015), and 10 µg/mL (*p* < 0.0001) (Table 1). Burst frequency and the duration of AP firing did not differ significantly between topiramate concentrations or compared to the Krebs buffer solution.

After FDR correction, only the reduction in AP number compared with baseline activity at all topiramate concentrations remained significant in myenteric neurons.

#### 2.5.2. DRG Neurons

The distribution of cluster response types following topiramate application was as follows: at a concentration of 150 µg/mL, topiramate induced no effect in 79.2% of clusters, an excitatory effect in 13.2%, an inhibitory effect in 5.7%, and a mixed effect in 1.9%. At 50 µg/mL, the distribution was 70.0% with no effect, 16.0% excitatory, 8.0% inhibitory, and 6.0% mixed. At 10 µg/mL, in 62.5% of clusters there was no change in neuronal activity in response to topiramate, in 25.0% of clusters an excitatory effect was recorded, in 6.3% an inhibitory effect, and in 6.3% a mixed effect (Figure 6b). No significant differences in the percentage of responding neurons, AP number, or burst frequencies between topiramate concentrations or compared to the Krebs buffer solution were recorded. In comparison with baseline activity, topiramate did not induce significant changes in AP number at any concentration (Table 1). The duration of AP firing was significantly shorter at 10 µg/mL compared to the Krebs buffer solution (*p* = 0.0249). No other significant differences were observed between Topiramate concentrations or compared to the Krebs buffer solution.

After FDR correction, none of the topiramate-induced effects remained significant in DRG neurons.

### 2.6. Summary of Functional Response Profiles

A summary of the functional response profiles of the investigated antiseizure drugs in myenteric and DRG neurons is provided in Table 2.

## 3. Discussion

This study represents a comparative functional neuropharmacological profiling of several ASDs commonly used in diseased dogs, in primary cultured guinea pig myenteric and DRG neurons. The tested drugs induced distinct drug- and neuron-type-specific response patterns. In myenteric neurons, topiramate produced the most pronounced suppression of neuronal excitability, whereas phenobarbital and levetiracetam caused only minor changes compared with buffer control. In contrast, most ASDs predominantly increased excitability in DRG neurons, while KBr exerted excitatory effects in both enteric and sensory neuronal populations. Together, these findings demonstrate drug-specific actions of clinically used ASDs on peripheral neurons and underscore fundamental functional differences between enteric and sensory neuronal systems.

These fundamental differences likely reflect the distinct anatomical organization and physiological roles of enteric versus sensory neurons, with myenteric neurons forming ganglionated intrinsic networks throughout the gastrointestinal tract that coordinate gastrointestinal reflex activity [13,14]. Enteric neuronal somata receive extensive synaptic input and serve as major sites of signal integration within these ganglia [15]. AP generation in enteric neurons predominantly depends on voltage-gated sodium (Na^+^) channels, with additional calcium (Ca^2+^) channel contribution to excitability and firing behavior [16]. In contrast, DRG neurons lack dendrites and do not receive classical synaptic input at the soma, and their excitability is modulated by non-synaptic mechanisms involving satellite glial cells and interactions with resident immune cells, particularly macrophages [9,17].

The most pronounced effect among all tested drugs in our experiment was elicited by topiramate in myenteric neurons. Topiramate induced a dose-dependent decrease in excitability, with nearly half of the clusters showing reduced activity at the highest concentration. Similarly, the number of APs fired per neuron significantly decreased following topiramate application across all concentrations. The observed changes could be mediated by several pharmacological mechanisms of action attributed to topiramate. One is the state-dependent inhibition of voltage-gated Na^+^ channels (VGSCs), stabilizing them in the open or inactivated state, and thereby reducing repetitive neuronal firing [18,19]. Myenteric neurons express VGSC subtypes Na_v_1.5 and Na_v_1.9, which contribute to the generation and propagation of APs [20,21]. Topiramate effectively inhibits Na_v_1.5 [21], and this mechanism is consistent with the reduced ability of myenteric neurons to generate APs observed in our experiments. In contrast, Na_v_1.8 and Na_v_1.9, which are tetrodotoxin-resistant [20] and poorly sensitive to classical ASDs, are unlikely primary targets of topiramate [19]. DRG neurons predominantly express Na_v_1.8 and Na_v_1.9 [20], rather than Naᵥ1.5. This channel expression profile may account for the comparatively weaker inhibitory effect of topiramate observed in this neuronal population.

Topiramate also antagonizes AMPA and kainate receptors, which are expressed in enteric neurons [22,23]. However, unlike in the CNS, in the ENS glutamate signaling is mediated almost exclusively through slow group I metabotropic glutamate receptors [24], while ionotropic glutamate receptor-mediated effects have also been reported in specific enteric pathways or neuronal subtypes [25]. Importantly, ionotropic glutamate receptors in the ENS do not universally generate classical excitatory postsynaptic potentials (EPSPs), but mediate fast EPSPs only in specific neuronal subtypes such as afterhyperpolarization neurons [26], which constitute a distinct subpopulation of enteric neurons. Accordingly, blockade of these receptors is less likely to produce a generalized inhibitory effect across enteric neurons. In contrast, in DRG neurons, AMPA and kainate receptors play an important role in pain pathways by regulating glutamate release at the central terminals of primary afferents and contributing to nociceptor sensitization [27,28]. However, these receptors are only minimally expressed on the DRG soma [29], which could explain the low inhibitory effect in our DRG neuronal culture experiments.

Another mechanism of topiramate is the blockade of voltage-gated Ca^2+^ channels (VGCCs), primarily affecting L-type and R-type channels [30,31], which are also present on enteric neurons [32]. L-type channels are strongly expressed on neuronal cell bodies and support somatic excitability as well as prolonged Ca^2+^ entry [33]. R-type Ca^2+^ channels are voltage-activated channels present in a large proportion of cultured myenteric neurons, contributing substantially to the somatodendritic Ca^2+^ current [34]. In addition, R-type channels support a Ca^2+^-dependent depolarizing phase during repolarization and can participate in fast excitatory synaptic signaling in specific subsets of enteric neurons [32,35]. In contrast, DRG neurons express predominantly N-type and R-type Ca^2+^ channels [22,36], which are located mainly at the central terminals of nociceptors [37]. Consequently, the comparatively minor effect of topiramate observed in DRG neurons is consistent with differences in VGCC subtype expression and their predominant compartmental distribution within the neuron, in contrast to the pronounced reduction in myenteric neuronal excitability observed in our experiments.

The action of topiramate on GABA receptors is unlikely to explain our findings. Although GABA_A_ receptors are expressed in both enteric and DRG neurons and their activation leads to depolarization due to the high intraneuronal Cl^−^ concentrations [38,39], we did not observe excitatory effects in our experiments, which is consistent with reports indicating that GABA_A_ modulation is not a major mechanism of action of topiramate [40].

Topiramate also inhibits carbonic anhydrases (CA), resulting in mild intracellular acidosis through reduced bicarbonate availability and altered Cl^−^/HCO_3_^−^ exchange [41]. Since CA activity is necessary for pH regulation in enteric neurons, this effect may partly account for the recorded inhibitory effect [42]. In contrast, the functional relevance of CA activity for DRG neurons remains less well defined [43].

Analyzing the effectiveness of ASDs, it should also be considered that antiseizure treatment is typically long-term, and that some drug–target interactions require sustained exposure. Previous studies have shown that the inhibitory effects of topiramate on voltage-gated Na^+^ and Ca^2+^ channels increase in a time- and activity-dependent manner [30,44]. Nevertheless, taken together, the observed decrease in myenteric neuronal activity is consistent with a synergistic mechanism involving topiramate’s effects on Na^+^ and Ca^2+^ channels, although direct confirmation of these channel-specific contributions was not performed in the present study.

Beyond its efficacy in epilepsy, topiramate has been associated with appetite reduction and weight loss [45,46] and has also been reported to reduce colonic damage in an experimental rodent model of intestinal inflammation [47]. In contrast to several ASDs that have been associated with increased appetite or weight gain [48], topiramate appears to have a distinct metabolic and gastrointestinal profile. The reduction in adiposity associated with topiramate is thought to be primarily mediated by central hypothalamic mechanisms, including reduced food intake [49]. However, appetite regulation also involves peripheral gastrointestinal, hormonal, and sensory signals that are integrated with central appetite circuits [50]. The pronounced inhibitory effect of topiramate observed in myenteric neurons suggests that enteric neuronal excitability should be considered in future studies on gut-related and appetite-related effects of topiramate. In addition, IBD-associated inflammation has been linked to enteric neuronal hyperexcitability [51], and together with experimental evidence suggesting beneficial effects of topiramate in IBD models, the inhibitory effect observed in myenteric neurons supports future investigation of topiramate in gastrointestinal disorders.

Consistent with our previous results, phenobarbital, one of the most effective and widely used ASDs in dogs, did not exert any effect on myenteric neuronal activity in the present study. The principal mechanism of action of phenobarbital is an allosteric modulation of GABA_A_ receptors, enhancing GABA-evoked Cl^−^ conductance. In enteric neurons, this would lead to Cl^−^ efflux and hence depolarization. Phenobarbital acts through barbiturate-sensitive modulation of GABA_A_ receptors, and its functional efficacy is influenced by receptor subunit composition [4,52]. Enteric neurons predominantly express α_1,2,3_ and α_5_ subunits [53], which exhibit slower gating kinetics and weaker barbiturate modulation [54], together explaining the low observed phenobarbital sensitivity. In addition, there is only limited GABAergic innervation within the ENS [55,56].

The effects of phenobarbital on Na^+^ and Ca^2+^ channels, as well as AMPA/kainate receptors, have been reported only at supratherapeutic concentrations [57,58,59], and are therefore considered negligible under the present experimental conditions. In DRG neurons, phenobarbital produced a clear excitatory effect, consistent with reports for related barbiturates across multiple species and in both adult and embryonic DRG cultures [60,61,62]. In DRG neurons, activation of GABA_A_ receptors also leads to membrane depolarization due to Cl^−^ efflux driven by high intracellular Cl^-^ concentrations [63]. This Cl^−^ gradient is maintained predominantly by the Na^+^-K^+^-2Cl^−^ cotransporter 1 (NKCC1) and, to a lesser extent, by the K^+^-Cl^−^ cotransporter 2 (KCC2), resulting in depolarizing GABA_A_ receptor-mediated responses [38]. Accordingly, the excitatory effect of phenobarbital observed in DRG neurons is consistent with its action on GABA_A_ receptors, although this mechanism was not directly tested in the present study.

In our experiments, KBr induced a strong excitatory effect on neuronal excitability in myenteric and DRG neurons. This effect can be partially explained by elevated extracellular K^+^, which depolarizes neurons and induces concentration-dependent increases in intracellular Na^+^ and Cl^−^, as previously shown in hippocampal and cortical neurons [64]. To isolate the bromide-specific component, KGlu was used as a K^+^-matched control with gluconate as the counterion. In direct comparison, KBr produced stronger excitation than KGlu in selected equimolar comparisons, suggesting a possible contribution of Br^−^ to the observed response. However, especially at the highest KBr/KGlu concentration, ionic and osmotic effects should also be considered.

Levetiracetam produced only minor changes in enteric neuronal activity but significantly increased firing activity in DRG neurons. Although the predominant mechanism of action of levetiracetam is its high-affinity binding to SV2A [65], additional mechanisms have been described [66], including suppression of AMPA receptor-mediated currents [67], weak modulation of GABA_A_ receptors [68], inhibition of N- and P/Q-type VGCCs [69,70] and modulation of Ca^2+^-activated K^+^ channels [71]. However, SV2A-dependent mechanisms are expected to play only a minor role under our in vitro conditions, where dissociated enteric neurons largely lose their intrinsic synaptic circuitry [13] and DRG somata lack functional synaptic contacts [17]. The excitatory effect on DRG neuronal activity could be explained by the inhibition of high-voltage-activated Ca^2+^ channels [72], diminishing the afterhyperpolarization via Ca^2+^-dependent K^+^ channels and thereby facilitating repetitive AP firing [73]. In addition, levetiracetam reduces the delayed-rectifier K^+^ current, preventing the neuronal membrane from fully returning to its resting potential and resulting in faster suprathreshold input. Furthermore, T-type Ca^2+^ channels, which are strongly excitatory in the soma of DRG neurons [36], are not inhibited by levetiracetam, preserving excitatory Ca^2+^ entry and hence contributing to the overall increase in neuronal excitability. Our findings are in contrast to those reported by Ozcan et al. [74], who demonstrated that acute application of levetiracetam in neonatal cultured DRG neurons induced membrane hyperpolarization, reduced repetitive AP firing, and inhibited depolarization-evoked Ca^2+^ transients, likely reflecting species differences and the immature electrophysiological properties of neonatal sensory neurons. As discussed earlier, enteric neurons depend on slightly different Ca^2+^ and K^+^ channels, explaining the missing effect recorded in our study.

Imepitoin is a relatively new ASD that is mainly used in dogs for the treatment of epilepsy [75] and noise phobia [76]. Its mechanism of action is based on modulation of GABA_A_ receptors; however, unlike classical ASDs, imepitoin binds to the benzodiazepine site and acts as a low-affinity, low-intrinsic-efficacy partial agonist, producing only 10–20% of the GABA potentiation observed with Diazepam [77]. Therefore, a weak functional effect is expected, which is consistent with our experiments, where imepitoin did induce altered activity in only single myenteric and DRG neuronal clusters. Although 1% DMSO increased AP number in DRG neurons, imepitoin at 100 µg/mL was associated with AP suppression relative to baseline. However, because the solvent control itself was bioactive, this finding cannot be attributed exclusively to imepitoin under the present formulation conditions and should be interpreted as preliminary.

In summary, the tested ASDs exerted distinct direct effects on myenteric and DRG neurons. Topiramate showed an enteric-predominant inhibitory profile, whereas KBr induced broad excitatory effects in both neuronal populations. Phenobarbital and levetiracetam showed more pronounced excitatory effects in DRG neurons than in myenteric neurons. Imepitoin produced only limited effects beyond the DMSO control, although high-concentration suppression was observed in DRG neurons relative to baseline. These findings provide a functional reference profile for future studies investigating FS-mediated effects in dogs treated with different ASDs and may help distinguish direct drug-induced neuronal effects from effects mediated by other fecal or microbiota-associated components.

From a translational perspective, these findings provide a functional overview of how different ASDs affect peripheral neuronal excitability in a concentration- and cell-type-dependent manner. This may be relevant for gastrointestinal disorders in which altered enteric neuronal excitability contributes to disease mechanisms, and may help identify drug-specific response profiles, particularly for topiramate and KBr, for future studies on peripheral neuronal modulation. In addition, because the ENS as well as DRG neurons are part of the gut–brain axis, ASD-induced changes in their neuronal activity may also be relevant for future studies on interactions between gastrointestinal and neurological or neurobehavioral disorders.

A limitation of the present study is that the tested concentrations were selected to represent estimated low, medium, and high exposure levels, corresponding to assumed distal intestinal, reported serum, and expected upper gastrointestinal concentrations, respectively. These levels are based on pharmacokinetic estimates and previous experimental data and therefore cannot fully reflect the dynamic exposure conditions occurring in vivo after oral administration. Accordingly, the present study should be considered a comparative functional profiling approach rather than a pharmacokinetic-pharmacodynamic model. For translational comparison, phenobarbital partly overlapped with the reported human therapeutic plasma range of 10–40 mg/L, whereas the medium and high concentrations of levetiracetam and topiramate exceeded commonly cited human reference ranges [78]. For KBr, no standard modern human therapeutic range is available; however, the lowest concentration was within the same order of magnitude as reported veterinary serum bromide concentrations, whereas higher concentrations should be interpreted as supra-therapeutic ionic/luminal exposure conditions [79]. Imepitoin is not used clinically in humans, and therefore no direct human therapeutic range comparison is available.

The measured osmolarity values of the working solutions should also be considered when interpreting the results. The highest KBr solution showed markedly increased osmolarity, whereas phenobarbital, topiramate, and levetiracetam showed negligible or very low values. However, these values refer to the solutions inside the application pipette and not necessarily to the osmolarity directly experienced by the neuronal clusters. Since the present study quantified rapid responses after a brief 500 ms application, the observed effects are unlikely to be explained solely by osmotic stress, as hypoosmolar stimulation of guinea pig enteric neurons was previously reported to induce delayed neuronal responses with maximal AP frequency approximately 10–12 s after application [80]. Nevertheless, transient osmotic, ionic, or pH-related contributions cannot be completely excluded, particularly at the highest KBr/KGlu concentration, because the pH of the working solutions was not measured.

Potential dye-related artifacts should also be considered. As with all voltage-sensitive dye recordings, photobleaching, phototoxicity, mechanical movement, and differences in dye loading or membrane staining may influence fluorescence signals. To limit these effects, recordings were kept short, standardized illumination and imaging settings were used, and analyses were based on relative fluorescence changes rather than absolute signal intensity.

In addition, IHC-linked responder-percentage analyses required successful IHC staining to assign cells to IHC-defined neuronal phenotypes and to determine the IHC-based denominator of total neurons per cluster. Therefore, cells with unsuccessful IHC could not be included in this specific IHC-linked responder-denominator analysis, even if electrophysiological AP firing could be detected. Incomplete IHC coverage may therefore have reduced the number of analyzable phenotype-assigned responder data points. However, it would not generate false electrophysiological responses, because AP firing was detected independently of IHC staining. To address this potential source of selection bias, unsuccessful IHC cases were manually re-checked and tabulated per compound, concentration, and cell type in Supplementary Table S3.

Another limitation is the hierarchical structure of the dataset, with neurons nested within clusters and clusters derived from independent cell cultures. Because the original dataset did not allow reliable retrospective reconstruction of the complete hierarchy for all endpoints and treatment conditions, a mixed-effects re-analysis could not be performed. To increase transparency, we report the available sample structure for each condition and applied Benjamini–Hochberg FDR correction to account for multiple comparisons.

Finally, guinea pig neuronal cultures were used as a surrogate model for drugs selected in a canine clinical context. Although guinea pig enteric neurons are a well-established model for functional studies of enteric neuronal excitability [81], species-specific differences in ion channel expression, GABA_A_ receptor composition, and sensory neuron populations may influence ASD responses [54,82]. The preparation of primary canine enteric or DRG neuronal cultures is associated with substantial technical and logistical challenges and was therefore beyond the scope of the present study [83]. Accordingly, the findings should be interpreted as comparative in vitro response profiles and not as direct evidence of ASD effects in canine neurons [82].

To verify the proposed mechanisms of action in future studies, specific channel blockers should be used to confirm or refine the hypothesized pathways or potentially reveal previously unknown mechanisms. Given the observed drug-specific effects on neuronal excitability, future studies should also evaluate other medications that may affect neuronal excitability, including antidepressants, which are used not only in patients with depression but also in those with epilepsy [84,85].

## 4. Materials and Methods

### 4.1. Animals and Cell Cultures

For the cell culture experiments, intestinal tissue and DRGs were obtained from 55 adult Dunkin Hartley guinea pigs of both sexes purchased from Charles River (France), aged 10–12 weeks, with an average body weight of 450 g. Guinea pigs were kept in the approved housing facility of the Institute for Physiology and Cell Biology at the University of Veterinary Medicine Hannover, Germany. Animals were housed in groups of 2 to 4 animals in cages with a size of 814 × 610 × 256 mm (L × W × H) and a floor area of 4000 cm^2^ (tecniplast GmbH, Hohenpeißenberg, Germany). Animals were kept under standardized conditions (20–24 °C room temperature, 60% humidity and a day/night cycle of 12:12 h) and received a pelleted standard diet (ssniff Spezialdiäten GmbH, Soest, Germany) and drinking water ad libitum. Fresh hay was provided daily. Guinea pigs were stunned by concussion and killed via exsanguination. All animal procedures were performed in accordance with approved institutional and national guidelines (see Ethics Statement). After killing of the animals, the small intestines and spinal column were removed, respectively. The intestine was placed into ice-cold carbogen-aerated (95% O_2_, 5% CO_2_) Krebs buffer solution with a stable pH of 7.4 (in mmol/L: 1.2 MgCl_2_, 2.5 CaCl_2_, 1.2 NaH_2_PO_4_, 117 NaCl, 25 NaHCO_3_, 11 glucose, 4.7 KCl). Primary myenteric and DRG neuronal cultures were prepared using previously described protocols [86,87]. For the myenteric neuronal cell culture, longitudinal muscle–myenteric plexus preparations were mechanically separated from the ileum, cut into small pieces approximately 1 × 1 mm, enzymatically digested, and 200 μL of the myenteric ganglia suspension was placed into cell culture dishes (Ibidi GmbH, Martinsried, Germany). Afterwards, the culture was incubated in medium M199 supplemented with 10% fetal bovine serum (FBS) (Gibco, Grand Island, NY, USA), 50–100 ng mL^−1^ mouse nerve growth factor 7S (Alomone Labs, Jerusalem, Israel), 5 mg mL^−1^ glucose, 100 U mL^−1^ penicillin, 100 mg mL−1 streptomycin (Gibco), and 2 mM arabinose-C-furanoside (Sigma-Aldrich Corporation, St. Louis, MO, USA).

The DRG neuronal cultures were prepared by isolating the DRG from the spinal column under a stereomicroscope and collecting them in a small glass bottle. The isolated ganglia were then digested using protease type I (Sigma-Aldrich, Steinheim, Germany), collagenase type II (516.2 U/mL; Gibco, Karlsruhe, Germany), and bovine serum albumin (BSA fraction V, 0.37%; Serva, Heidelberg, Germany) for 40–50 min. Following several washing steps with ice-cold sterile Krebs buffer solution, the resulting pellet was resuspended in 600–1200 μL of culture medium (M199 GlutaMAX; Gibco) supplemented with 1% penicillin–streptomycin (Gibco), 30 mM glucose, 50 ng/mL mouse nerve growth factor 7S (Alomone Labs, Jerusalem, Israel), and 10% fetal bovine serum (Gibco). Subsequently, 300 μL of the ganglia suspension was placed into each culture dish, resulting in four to eight dishes per preparation (Ibidi dish 35 mm with IbiTreat coating; Ibidi, Martinsried, Germany).

Both myenteric and DRG neurons were cultured under standard culture conditions (5% CO_2_, 37 °C, 95% humidity) for 11–16 days to acquire ganglion-like interconnected neuronal clusters. The medium was changed every 2–3 days.

### 4.2. Antiseizure Drugs

The ASDs selected for this study were those commonly used in the treatment of canine epilepsy, including phenobarbital, KBr, levetiracetam, imepitoin, and topiramate. For each drug, three concentration levels were used, defined as low, medium, and high (Table 3). Based on half-life and bioavailability, estimated drug concentrations in different segments of the gastrointestinal tract were calculated. For the selection of the low concentration, we additionally considered data from our previous study, in which the concentration of phenobarbital detected in FS from dogs receiving standard treatment was 3.6 μg/mL, which is lower than typical therapeutic serum levels [12]. The medium concentration was selected based on reported serum concentrations in treated dogs. The high concentration was selected to approximate the estimated higher luminal drug concentration expected in the upper gastrointestinal tract before progressive absorption and transit to more distal intestinal segments [88]. Thus, the three selected concentrations were intended to represent assumed distal intestinal, systemic, and upper gastrointestinal exposure levels, respectively.

Additionally, control experiments were conducted using the respective vehicle solutions. Except for imepitoin and KBr, Krebs buffer solution was used as the control for all ASDs. Since imepitoin was dissolved in 1% DMSO, 1% DMSO was used as the corresponding vehicle control to exclude solvent-related effects. Due to the known excitatory effects of K^+^ ions on neurons, KGlu was used as a control for KBr, with concentrations calculated according to the respective molecular weights. Based on this calculation, 100 mg/mL KBr provided the same K^+^ concentration as 840 mmol/L KGlu, 10 mg/mL KBr corresponded to 84 mmol/L KGlu, and 1 mg/mL KBr corresponded to 8.4 mmol/L KGlu. The osmolarity of available working solutions was measured after dilution. The highest KBr solution showed a measured osmolarity of approximately 1450 mOsm/kg, whereas the osmolarities of phenobarbital, topiramate, and levetiracetam working solutions were negligible or very low (0–2 mOsm/kg).

For most experiments, all drugs were freshly diluted in distilled water immediately before use, except for imepitoin, which was dissolved in 1% DMSO. For KBr and KGlu, diluted working solutions were prepared from stock solutions stored for up to six months due to their known stability in diluted form [89].

### 4.3. Neuroimaging

For each investigated drug, a minimum of three dishes from three different cell cultures, from both myenteric and DRG neurons, were analyzed. The number of clusters per dish varied depending on cell viability, but drugs were applied to at least seven clusters per dish. Clusters were defined as an accumulation of at least three neurons in close proximity. A blank recording was made without any stimulus to evaluate spontaneous (baseline) neuronal activity. Afterwards, the respective ASD was applied to single neuronal clusters for 500 ms by local pressure application (PDES-2lL; npi electronic GmbH, Tamm, Germany). Drug concentrations refer to the nominal concentrations in the application pipette. Approximately 2 µL were delivered over 500 ms into a continuously perfused recording chamber containing approximately 2 mL Krebs solution. Thus, the actual concentration and osmolarity reaching the neuronal cluster were not directly measured and may have been lower than the nominal pipette values due to local dilution. The application was performed with a 200 ms delay and the total recording time was 1.6 s. To record the AP discharge, the voltage-sensitive dye 1-(3-sulfanato-propyl)-4-[b-[2-(di-n-octylamino)-6-naphtyl] vinyl] pyridinium betaine (Di-8-ANEPPS, Thermo Fisher Scientific) was used to stain the neurons for 12 min at room temperature. In previous studies, it has been shown that the staining technique does not influence the electrophysiological properties of the neurons [90]. Di-8-ANEPPS was used as a fast-response, membrane-bound potentiometric dye to monitor relative changes in membrane potential. Therefore, no calibration to absolute membrane voltage values was performed. Optical recordings were evaluated as relative fluorescence changes (ΔF/F), following previously established enteric neuroimaging approaches [91]. Representative bright-field/phase-contrast and Di-8-ANEPPS fluorescence images illustrating the neuronal culture preparation and staining quality are shown in Figure 1 and Figure 2. After staining, the dishes were placed into a dish holder on an inverted epifluorescence microscope (Olympus IX71; Olympus Corporation, Hamburg, Germany). The dishes were continuously perfused with Krebs buffer solution (37 °C, pH 7.4) containing (in mM): 1.2 MgCl_2_, 2.5 CaCl_2_, 117 NaCl, 15.0 NaHCO_3_, 4.7 KCl, and 11 glucose, at a rate of 10 mL/min throughout the experiment. To detect neuronal activity, the clusters were excited using light emitted by a green high-power LED (LET A2A true green, 521 nm, 700 mA; OSRAM GmbH, Munich, Germany) in combination with a filter set containing a 525/15 nm bandpass excitation filter (AHF Analysentechnik AG, Tübingen, Germany), a dichroic mirror with a separation wavelength of 565 nm, and a bandpass filter with a spectrum of 560/15 nm (AHF Analysentechnik AG). A 40× oil-immersion objective lens (UApo 40× OI3/340 Oil NA 1.35–0.5; Olympus Corporation) was used to achieve the required high light intensity and an appropriate signal-to-noise ratio. Changes in Di-8-ANEPPS fluorescence intensity were detected using a high-speed complementary metal-oxide-semiconductor (CMOS) camera with a 1.25 kHz frame rate and a spatial resolution of 256 × 256 pixels (DaVinci1K, RedShirt Imaging, LLC, Decatur, GA, USA). Combined with the 40× oil-immersion objective lens, this resulted in a spatial resolution of 2.2 μm^2^ per pixel. The electrical activity of the neurons was registered by the software TurboSM 64 (RedShirt Imaging LLC, Decatur, GA, USA; https://redshirtimaging.com, accessed on 12 August 2026).

### 4.4. Immunohistochemistry

Immunohistochemistry (IHC) was performed to count the total number of neurons per cluster by staining neuron-specific enolase (NSE). The cultured neurons in the dishes were fixed immediately after the neuroimaging experiments for 15 min at room temperature in a solution containing 4% paraformaldehyde and 0.002% picric acid (Sigma-Aldrich Corporation). Afterwards, tissues were washed three times (10 min each) in phosphate-buffered saline (PBS) and pre-incubated for 1 h in PBS containing 4% horse serum (Sigma-Aldrich Corporation) and 0.5% Triton X-100 (Sigma-Aldrich Corporation). The cultures were then incubated for 12 h at room temperature in a solution containing the primary antibody (rabbit anti-NSE, 1:8000, Polysciences, Inc., Warrington, PA, USA), washed three times in PBS, and incubated for 2 h in a solution containing the secondary antibody (Cy3-conjugated donkey anti-rabbit IgG, 1:500, from Dianova GmbH, Hamburg, Germany). In the final step, cultures were washed three times in PBS and covered with a solution of PBS (pH 7.0) containing 0.1% NaN3 and 80% glycerol (Sigma-Aldrich Corporation). Stained cultures were examined using an epifluorescence microscope with appropriate filters. Images were taken and analyzed with a monochrome camera (XM 10; Olympus Corporation) combined with Olympus cellSens Standard Software v4.5 (Olympus Corporation, Tokyo, Japan). A representative NSE immunostaining image used for neuronal identification and counting is provided in Supplementary Figure S14.

### 4.5. Data Analysis and Statistics

Raw data of neuroimaging experiments were analyzed using TurboSM 64 software (RedShirt Imaging LLC; https://redshirtimaging.com, accessed on 12 August 2026). Further data analysis and graphic visualization were performed using Microsoft Excel for Mac (version 16.78.3, Redmond, WA, USA) and GraphPad Prism (Prism 10, Version 10.3.1, San Diego, CA, USA). The counting of neurons after IHC was performed using ImageJ version 1.8.0 (Wayne Rasband, National Institutes of Health, Bethesda, MD, USA).

TurboSM 64 software was used to calculate the number of APs of individual neurons within each cluster. For signal quantification, optical traces were analyzed as baseline-normalized relative fluorescence changes (ΔF/F). A representative trace of a myenteric and DRG neuron responding to KBr application is depicted in Figure 3. APs were counted from transient fluorescence deflections detected in individual neuronal regions of interest within each cluster, using identical acquisition and analysis criteria across recordings. Initially, we analyzed drug-induced changes in neuronal activity at the cluster level. The effects of the drug on neuronal activity in clusters were consequently divided into four groups. An excitatory effect was defined when the drug increased neuronal excitability in one or more neurons within the cluster; thus, the number of APs after application was higher than baseline. An inhibitory effect was defined when the drug decreased neuronal excitability in one or more neurons within the cluster, indicated by a lower number of APs after application compared to baseline. A mixed effect was defined when both excitatory and inhibitory effects occurred within the same cluster, meaning that the number of APs increased in some neurons but decreased in others after application, respectively. No effect was defined when the application of the drug did not lead to a change in the number of APs elicited by the neurons. Thus, for all clusters, the distribution of induced effects of each drug was calculated and expressed as the percentage of clusters.

Subsequently, the following neuronal characteristics were evaluated:

- The percentage of responding neurons within a cluster relative to the total number of neurons counted by IHC.
- The number of APs elicited by each neuron was counted before and after drug application. Responders were defined as neurons with a greater AP discharge after drug application compared to baseline activity.
- The change in AP number (ΔAP number) was calculated for each analyzed neuron as the AP number after drug application minus the AP number during baseline activity.
- Burst frequency was calculated as the ratio of the number of APs to the total duration of AP discharge after drug application.
- AP firing duration was defined as the time interval between the first and the last AP.

Burst frequency and AP number were analyzed as distinct parameters: AP number reflects the total number of detected action potentials, whereas burst frequency relates the number of fired APs to the duration of the active discharge period.

Data were analyzed for normal distribution using the Shapiro–Wilk test. For datasets conforming to a Gaussian distribution, group comparisons were performed using ordinary one-way ANOVA followed by Tukey’s post hoc test, corrected for multiple comparisons. For non-Gaussian datasets, the Kruskal–Wallis test was applied, followed by Dunn’s post hoc test, corrected for multiple comparisons, for pairwise group analysis. For paired comparisons before and after drug application, either the Wilcoxon matched-pairs signed-rank test or paired t-test was used, depending on data distribution. Nominal *p*-values of <0.05 were considered statistically significant before additional FDR correction.

Intergroup comparisons of KBr and KGlu were performed to distinguish drug-specific from potassium-related effects. Statistical analysis was performed using Kruskal–Wallis tests with Dunn’s post hoc analysis. All concentrations were compared, but only equimolar K^+^ concentrations were evaluated in detail.

To address multiplicity, *p*-values from the planned comparisons were additionally adjusted during revision using the FDR procedure. FDR correction was applied separately within each drug family across the corresponding test family. Before–after comparisons assessing changes in AP number after drug application were corrected separately within each drug family. Findings that remained significant after FDR correction are indicated in the Results section.

For each experimental condition, the number of responder cells, recorded clusters, and independent primary cell cultures is reported in Table S2. Depending on the endpoint, analyses were based on responder cells or recorded neuronal clusters, whereas independent primary cell cultures represented the biological level of replication. In total, cultures were prepared from 24 animals for myenteric neurons and 16 animals for DRG neurons.

No formal a priori power calculation was performed. Sample size was determined by the availability of successfully established primary neuronal cultures and technically usable recordings. The study was designed as an exploratory in vitro comparison of ASD-induced neuronal response profiles rather than as a confirmatory efficacy study. To account for biological variability, experiments were performed using three independent biological replicates, i.e., primary neuronal cultures prepared from three different animals. This approach was chosen to capture inter-animal biological variation while adhering to the principles of reduction under the 3Rs framework. Within each biological replicate, multiple technically successful recordings were obtained and analyzed.

## Figures and Tables

**Table 1:** Effects of antiseizure drugs on neuronal action potential discharge in myenteric and dorsal root ganglion (DRG) neurons.

Compound	Low	Medium	High	Low	Medium	High
	Myenteric			DRG		
Phenobarbital	−2	−1	−1	+3.5	+3	+4
Potassium bromide	+3	+4	+5	+2	+5	+7
Potassium gluconate	0	+4	+7	+4	+5	+8
Imepitoin	−1	−2	−1	+2	+2	−7
Levetiracetam	−3	−2	−1	+2	+5	+5
Topiramate	−2	−1	−3	+2	+2	+2
Dimethyl sulfoxide 1% *		−1			+3	
Krebs buffer *		+1			−2	

* Were tested at a single concentration (not corresponding to medium concentration). Values represent the median change in action potential (AP) number after drug application relative to baseline activity in myenteric and DRG neurons. Low, medium, and high indicate the tested antiseizure drug (ASD) concentrations. ΔAP number was calculated for each analyzed neuron as the number of APs after drug application minus the number of APs during baseline activity. Positive values (+) indicate increased AP firing after application, whereas negative values (−) indicate reduced AP firing. Detailed values including median ΔAP number, interquartile range (IQR), and N of analyzed neurons for each condition are provided in Supplementary Table S1.

**Table 2:** Summary of the effects of antiseizure drugs on excitability of cultured primary enteric and DRG neurons.

Drug	Myenteric Neurons	DRG Neurons
Phenobarbital	Minor/inconsistent	Predominantly excitatory
KBr	Excitatory	Excitatory
Imepitoin	No clear effect vs. DMSO	Suppression at high concentration
Levetiracetam	Mild inhibitory effect	Predominantly excitatory
Topiramate	Predominantly inhibitory	Minor/limited

Profiles are based on the significant effects described in the results section. DRG, dorsal root ganglion; KBr, potassium bromide; DMSO, dimethyl sulfoxide.

**Table 3:** Tested antiseizure drugs with controls and concentrations.

Compound	Low Concentration	Medium Concentration	High Concentration
Phenobarbital	3.6 μg/mL	16.8 μg/mL	40 μg/mL
Potassium bromide	1 mg/mL	10 mg/mL	100 mg/mL
Imepitoin	10 μg/mL	50 μg/mL	100 μg/mL
Levetiracetam	10 μg/mL	50 μg/mL	100 μg/mL
Topiramate	10 μg/mL	50 μg/mL	150 μg/mL
Potassium gluconate	8.4 mmol/L	84 mmol/L	840 mmol/L
Dimethyl sulfoxide 1%		constant concentration	
Krebs Buffer solution		constant concentration	

Concentrations are reported in mass-based or molar units depending on compound solubility and experimental design. Potassium gluconate concentrations are given in mmol/L to allow for equimolar comparison with potassium bromide. Control solutions were applied at a constant concentration.

## Data Availability

The raw data supporting the conclusions of this article will be made available by the authors upon request.

## Supplementary Materials

The following supporting information can be downloaded at https://www.mdpi.com/article/10.3390/ph19091356/s1: Figure S1: Potassium gluconate (KGlu)-induced effects on neuronal excitability at the cluster level in myenteric and dorsal root ganglion (DRG) neuronal cultures; Figure S2: Effects of potassium gluconate (KGlu) on myenteric neuronal excitability; Figure S3: Effects of potassium gluconate (KGlu) on dorsal root ganglion (DRG) neuronal excitability; Figure S4: Effects of potassium gluconate (KGlu) on dorsal root ganglion (DRG) neuronal excitability; Figure S5: Phenobarbital-induced effects on neuronal excitability at the cluster level in myenteric and dorsal root ganglion (DRG) neuronal cultures; Figure S6: Imepitoin-induced effects on neuronal excitability at the cluster level in myenteric and dorsal root ganglion (DRG) neuronal cultures; Figure S7: Levetiracetam-induced effects on neuronal excitability at the cluster level in myenteric and dorsal root ganglion (DRG) neuronal cultures; Figure S8: Effects of buffer and DMSO (1%) on neuronal excitability at the cluster level in myenteric and dorsal root ganglion (DRG) neuronal cultures; Figure S9: Effects of Phenobarbital (Pb) on dorsal root ganglion (DRG) neuronal excitability; Figure S10: Effects of potassium bromide (KBr) on myenteric neuronal excitability; Figure S11: Effects of potassium bromide (KBr) on dorsal root ganglion (DRG) neuronal excitability; Figure S12: Comparison of drug-induced effects on neuronal excitability following application of potassium bromide (KBr) and potassium gluconate (KGlu) at equimolar potassium concentrations; Figure S13: Effects of Levetiracetam (Lev) on dorsal root ganglion (DRG) neuronal excitability; Figure S14: Representative NSE immunostaining of cultured myenteric neurons; Table S1: Median change in action potential number (ΔAP number) after drug application relative to baseline activity; Table S2: Sample structure for responder-cell, recorded-cluster, and independent-culture levels across experimental conditions; Table S3: Unsuccessful IHC cases and actively responding cells per experimental condition.

## Abbreviations

The following abbreviations are used in this manuscript:

AMPA	α-amino-3-hydroxy-5-methyl-4-isoxazolepropionic acid
AP	action potential
ASD	antiseizure drug
CA	carbonic anhydrase
Ca^2+^	calcium
Cl^−^	chloride
CNS	central nervous system
DRG	dorsal root ganglion
ENS	enteric nervous system
FS	fecal supernatant
GABA	γ-aminobutyric acid
IBD	inflammatory bowel disease
IE	idiopathic epilepsy
K^+^	potassium
KBr	potassium bromide
KGlu	potassium gluconate
Na^+^	sodium
VGCC	voltage-gated calcium channel
VGSC	voltage-gated sodium channel
