# Supplementary material for: Comparative Neuropharmacological Effects of Antiseizure Drugs on Cultured Myenteric and Dorsal Root Ganglion Neurons
Source: Pharmaceuticals (Basel). 2026 Aug 27;19(9):1356. doi: 10.3390/ph19091356 (PMC13610870; doi:10.3390/ph19091356)
Supplement: Supplementary file 1 [file pharmaceuticals-19-01356-s001.zip › pharmaceuticals-4342144-supplementary.pdf]

Supplementary Materials

1.1 Potassium Gluconate (KGlu, Potassium control)

1.1.1 Myenteric neurons

The distribution of cluster response types following KGlu application was as follows: at a concentration of 840 mmol/L, KGlu induced excitatory effects on neuronal excitability in 75.7% of clusters, no effect in 18.9%, and a mixed effect in 5.4%. At 84 mmol/L, the distribution was 66.7% excitatory, 11.1% no effect, and 22.2% mixed effect. At 8.4 mmol/L, in 35.3% of clusters no effect was recorded, in 25.5% an excitatory effect, in 23.5% an inhibitory effect, and in 15.7% a mixed effect (Figure S1).

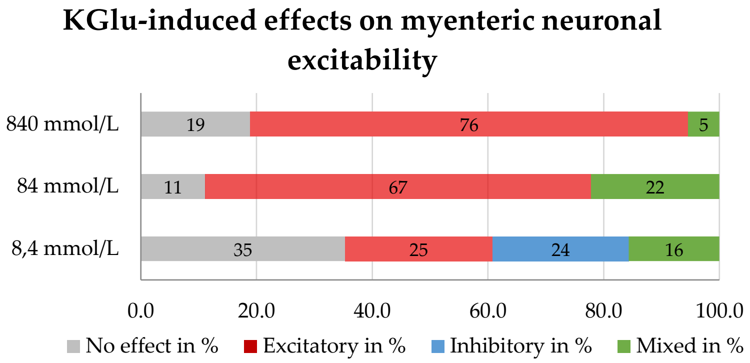

(**a**)

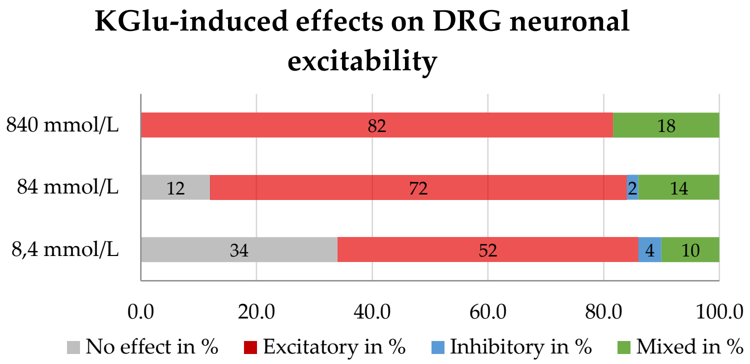

(**b**)

Figure S1. Potassium gluconate (KGlu)-induced effects on neuronal excitability at the cluster level in myenteric (**a**) and dorsal root ganglion (DRG) neuronal cultures (**b**). Stacked horizontal bar charts show the proportion of neuronal clusters, in which application of KGlu induced no effect (grey), excitatory (red), inhibitory (blue), or mixed excitatory/inhibitory effects (green) on neuronal excitability at concentrations 840, 84 and 8.4 mmol/L.

KGlu induced responses in a significantly greater proportion of neurons per cluster at 840 mmol/L compared with 8.4 mmol/L ($p = 0.0027$) and Krebs buffer solution ($p = 0.0008$), and at 84 mmol/L compared with 8.4 mmol/L ($p = 0.0062$) and Krebs buffer solution ($p = 0.0017$) (Figure S2a).

KGlu induced a significantly higher number of APs at 840 mmol/L compared with 84 mmol/L ($p = 0.0021$), 8.4 mmol/L ($p < 0.0001$), and Krebs buffer solution ($p = 0.0013$). In addition, 84 mmol/L induced significantly more APs than 8.4 mmol/L ($p = 0.0036$) (Figure S2b). Compared with baseline activity, KGlu induced a significant increase in AP number

at 840 mmol/L and 84 mmol/L (both $p < 0.0001$), whereas 8.4 mmol/L did not induce a significant change ($p = 0.3683$) (Table 1).

KGlu induced a significantly higher burst frequency at 840 mmol/L compared with Krebs buffer solution ($p = 0.0057$). In addition, 84 mmol/L also induced a significantly higher burst frequency compared with Krebs buffer solution ($p = 0.0398$) (Figure S2c). No significant differences in AP duration were observed between KGlu concentrations or compared with Krebs buffer solution.

After FDR correction, the following KGlu effects remained significant in myenteric neurons: responder proportion at 840 mmol/L vs. 8.4 mmol/L and Krebs buffer, and at 84 mmol/L vs. 8.4 mmol/L and Krebs buffer; AP number at 840 mmol/L vs. 84 mmol/L, 8.4 mmol/L, and Krebs buffer, and at 84 mmol/L vs. 8.4 mmol/L; AP number vs. baseline at 840 mmol/L and 84 mmol/L; and burst frequency at 840 mmol/L vs. Krebs buffer. The initially significant increase in burst frequency at 84 mmol/L vs. Krebs buffer did not remain significant after FDR correction.

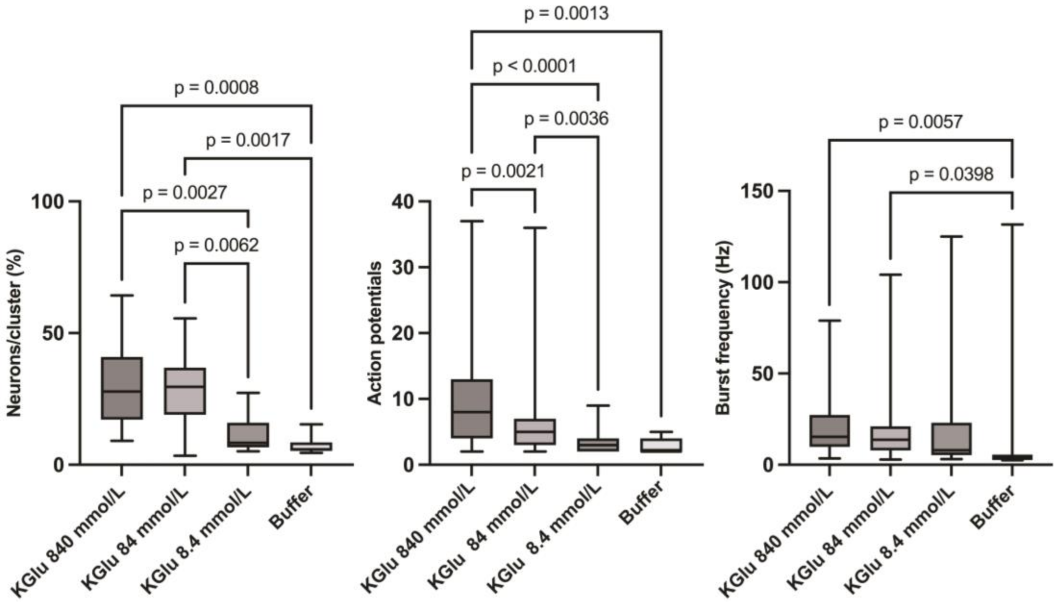

(**a**) (**b**) (**c**)

Figure S2. Effects of potassium gluconate (KGlu) on myenteric neuronal excitability. Data are shown as box-and-whisker plots (median, interquartile range, minimum-maximum). Significant differences between conditions, including comparisons between concentrations and buffer, are indicated (Dunn's multiple comparisons test; p-values shown). (**a**) Myenteric neurons: neurons per cluster (%). Percentage of myenteric neurons per cluster exhibiting drug-induced changes in activity following application of KGlu at 840, 84, 8.4 mmol/L and Krebs buffer solution. (**b**) Myenteric neurons: action potential number. Number of action potentials recorded in myenteric neurons following application of KGlu at 840, 84, and 8.4 mmol/L and Krebs buffer solution. (**c**) Myenteric neurons: burst frequency (Hz). Burst frequency of myenteric neurons following application of KGlu at 840, 84, 8.4 mmol/L and Krebs buffer solution.

5.1.2 DRG neurons

The distribution of cluster response types following KGlu application was as follows: at a concentration of 840 mmol/L, KGlu induced excitatory effects in 81.6% of clusters, and mixed effects in 18.4%. At 84 mmol/L, the distribution was 72.0% excitatory, 12.0% with no effect, 14.0% mixed effect, and in 2.0% an inhibitory effect. At 8.4 mmol/L, in 52.0% an excitatory effect, in 34% of clusters no effect was recorded, in 10.0% a mixed effect, and in 4.0% an inhibitory effect (Figure S1).

KGlu induced responses in a significantly greater proportion of neurons per cluster at 840 mmol/L compared with 84 mmol/L ($p < 0.0001$), 8.4 mmol/L ($p <0.0001$), and Krebs buffer solution ($p =0.0001$) (Figure S3a).

KGlu at 840 mmol/L induced a significantly higher number of APs compared with 84 mmol/L ($p < 0.0001$), 8.4 mmol/L ($p < 0.0001$), and Krebs buffer solution ($p = 0.0158$). In addition, KGlu at 84 mmol/L induced a significantly higher number of APs than 8.4 mmol/L ($p = 0.0085$) (Figure S3b). Compared with baseline activity, KGlu induced a significant increase in AP number at 840 mmol/L ($p < 0.0001$), 84 mmol/L ($p < 0.0001$), and 8.4 mmol/L ($p < 0.0001$) in DRG neurons (Table 1).

KGlu at 840 mmol/L induced a significantly higher burst frequency compared with 84 mmol/L ($p < 0.0001$), 8.4 mmol/L ($p < 0.0001$), and Krebs buffer solution ($p = 0.0007$). In addition, KGlu at 84 mmol/L induced a significantly higher burst frequency than Krebs buffer solution ($p = 0.0487$), whereas no significant difference was detected between 84 mmol/L and 8.4 mmol/L (Figure S4a).

KGlu at 840 mmol/L induced a significantly longer AP duration compared with 84 mmol/L ($p < 0.0001$). In contrast, KGlu at 84 mmol/L induced a significantly shorter AP duration compared with 8.4 mmol/L ($p = 0.0008$) and Krebs buffer solution ($p = 0.0023$) (Figure S4b).

After FDR correction, the following KGlu effects remained significant in DRG neurons: responder proportion at 840 mmol/L vs. 84 mmol/L, 8.4 mmol/L, and Krebs buffer; AP number at 840 mmol/L vs. 84 mmol/L, 8.4 mmol/L, and Krebs buffer, and at 84 mmol/L vs. 8.4 mmol/L; AP number vs. baseline at all three KGlu concentrations; burst frequency at 840 mmol/L vs. 84 mmol/L, 8.4 mmol/L, and Krebs buffer; and AP firing duration at 840 mmol/L vs. 84 mmol/L, and at 84 mmol/L vs. 8.4 mmol/L and Krebs buffer. The initially significant increase in burst frequency at 84 mmol/L vs. Krebs buffer did not remain significant after FDR correction.

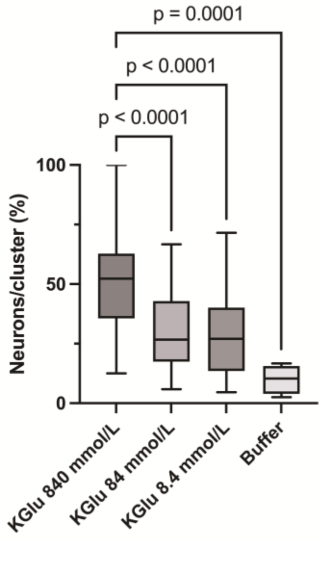

(**a**)

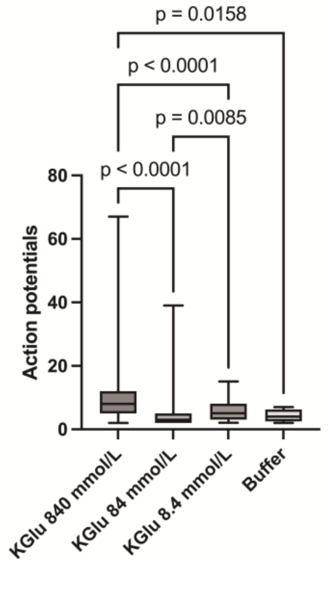

(**b**)

Figure S3. Effects of potassium gluconate (KGlu) on dorsal root ganglion (DRG) neuronal excitability. Data are shown as box-and-whisker plots (median, interquartile range, minimum–maximum). Significant differences between conditions, including comparisons between concentrations and buffer, are indicated (Tukey's test for panel a; Dunn's test for panel b; p-values shown). (**a**) DRG neurons: neurons per cluster **(%).** Percentage of DRG neurons per cluster exhibiting drug-induced changes in activity following application of KGlu at 840, 84, and 8.4 mmol/L and Krebs buffer

solution. (**b**) DRG neurons: action potential number. Number of action potentials recorded in DRG neurons following application of KGlu at 840, 84, and 8.4 mmol/L and Krebs buffer solution.

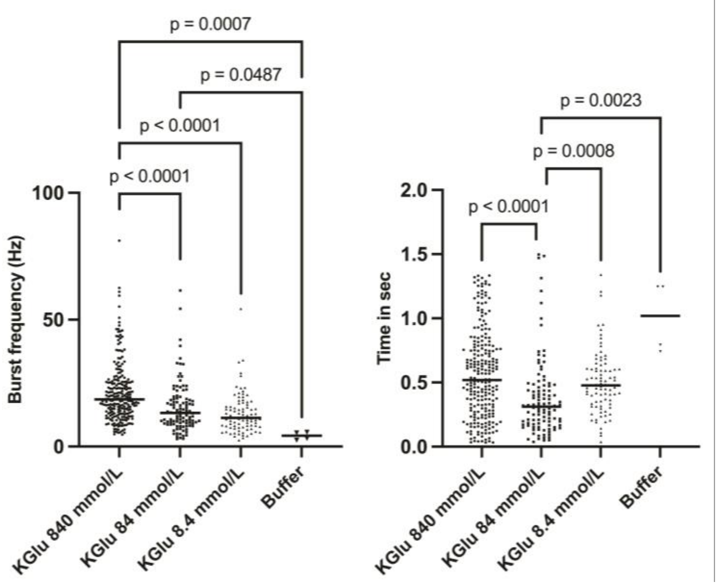

(**a**) (**b**)

Figure S4. Effects of potassium gluconate (KGlu) on dorsal root ganglion (DRG) neuronal excitability. Data are shown as scatter plots, with median values indicated. Significant differences between conditions, including comparisons between concentrations and buffer, are indicated (Dunn's multiple comparisons test; p-values shown). (**a**) DRG neurons: burst frequency (Hz). Burst frequency of DRG neurons following application of KGlu at 840, 84, and 8.4 mmol/L and Krebs buffer solution. (**b**) DRG neurons: duration of action potential firing (s). Duration of action potential firing in DRG neurons following application of KGlu at 840, 84, and 8.4 mmol/L and Krebs buffer solution.

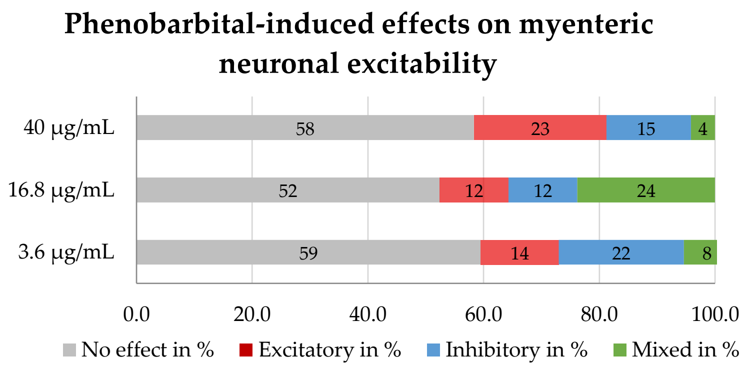

(**a**)

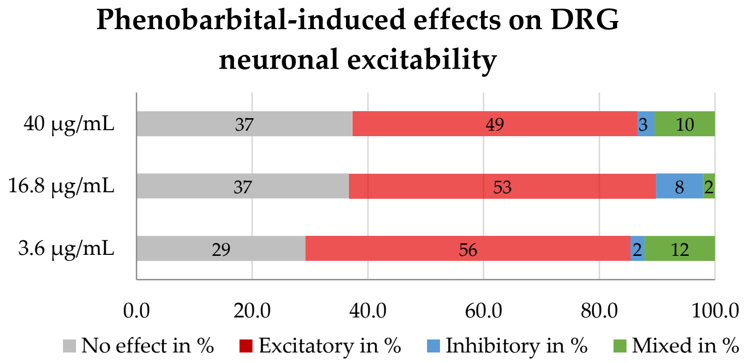

(**b**)

Figure S5. Phenobarbital-induced effects on neuronal excitability at the cluster level in myenteric (**a**) and dorsal root ganglion (DRG) neuronal cultures (**b**). Stacked horizontal bar charts show the proportion of neuronal clusters, in which application of Phenobarbital induced no effect (grey), excitatory (red), inhibitory (blue), or mixed excitatory/inhibitory effects (green) on neuronal excitability at concentrations 40, 16.8, and 3.6 μg/mL.

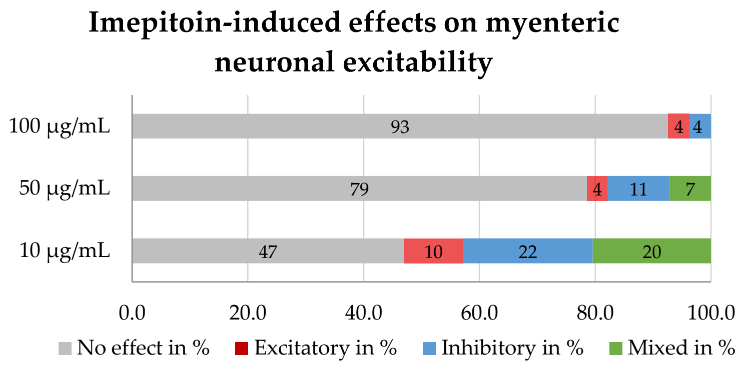

(**a**)

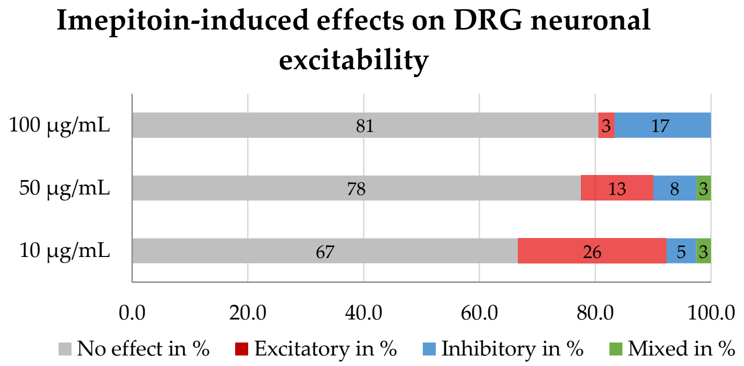

(**b**)

Figure S6. Imepitoin-induced effects on neuronal excitability at the cluster level in myenteric (**a**) and dorsal root ganglion (DRG) neuronal cultures (**b**). Stacked horizontal bar charts show the proportion of neuronal clusters, in which application of Imepitoin induced no effect (grey), excitatory (red), inhibitory (blue), or mixed excitatory/inhibitory effects (green) on neuronal excitability at concentrations 100, 50, and 10 μg/mL.

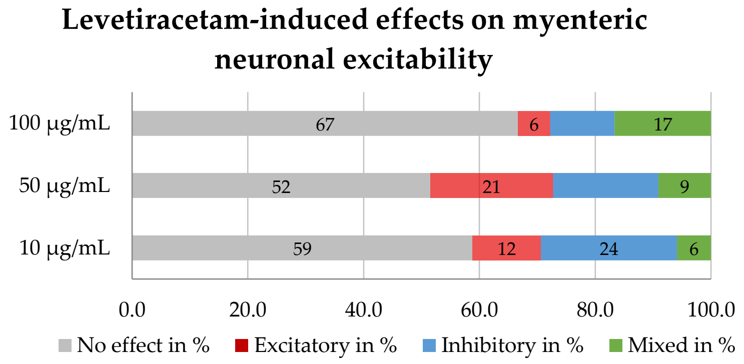

(**a**)

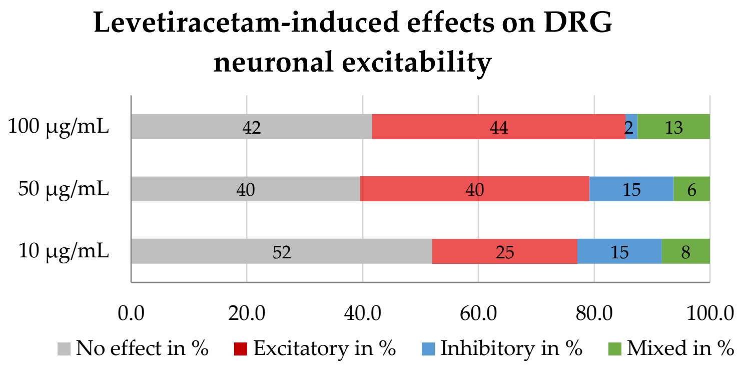

(**b**)

Figure S7. Levetiracetam-induced effects on neuronal excitability at the cluster level in myenteric (**a**) and dorsal root ganglion (DRG) neuronal cultures (**b**). Stacked horizontal bar charts show the proportion of neuronal clusters, in which application of Levetiracetam induced no effect (grey), excitatory (red), inhibitory (blue), or mixed excitatory/inhibitory effects (green) on neuronal excitability at concentrations 100, 50, and 10 µg/mL

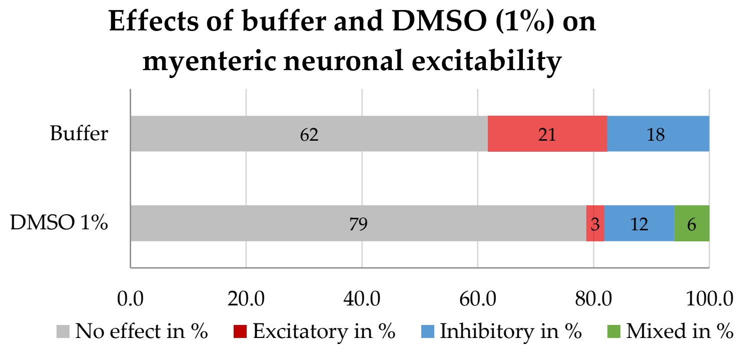

(**a**)

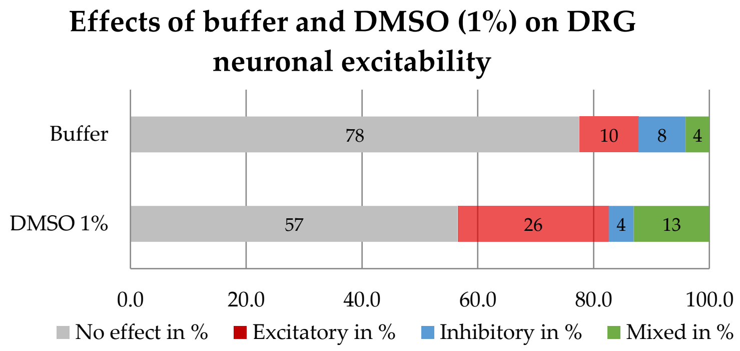

(**b**)

Figure S8. Effects of buffer and DMSO (1%) on neuronal excitability at the cluster level in myenteric (**a**) and dorsal root ganglion (DRG) neuronal cultures (**b**). Stacked horizontal bar charts show the proportion of neuronal clusters, in which application of buffer or DMSO (1%) induced no effect (grey), excitatory (red), inhibitory (blue), or mixed excitatory/inhibitory effects (green) on neuronal excitability.

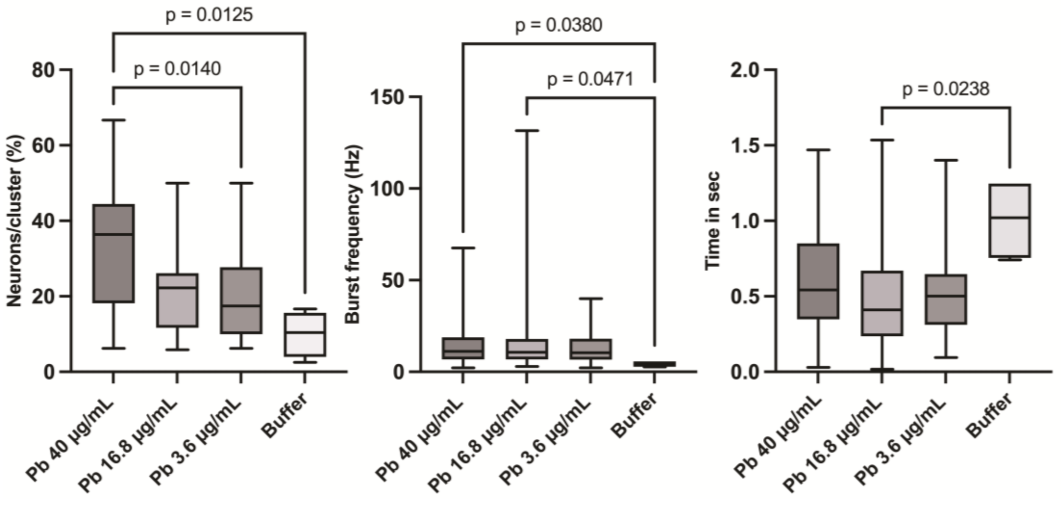

(**a**) (**b**) (**c**)

Figure S9. Effects of Phenobarbital (Pb) on dorsal root ganglion (DRG) neuronal excitability. Data are shown as box-and-whisker plots (median, interquartile range, minimum–maximum). Significant differences between conditions, including comparisons between concentrations and buffer, are indicated (Dunn's multiple comparisons test; p-values shown). (**a**) DRG neurons: neurons per cluster (%). Percentage of DRG neurons per cluster exhibiting drug-induced changes in activity following application of Phenobarbital at 40, 16.8, and 3.6 µg/mL and Krebs buffer solution. (**b**) DRG neurons: burst frequency (Hz). Burst frequency of DRG neurons following application of Phenobarbital at 40, 16.8, and 3.6 µg/mL and Krebs buffer solution. (**c**) DRG neurons: duration of neuronal activity (s). Duration of neuronal activity in DRG neurons following application of Phenobarbital at 40, 16.8, and 3.6 µg/mL and Krebs buffer solution.

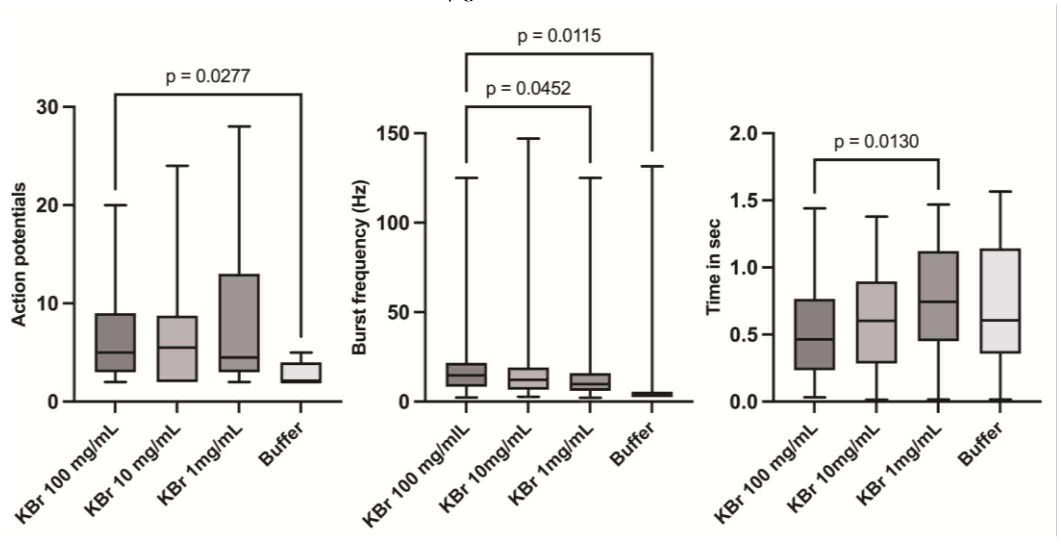

(**a**) (**b**) (**c**)

Figure S10. Effects of potassium bromide (KBr) on myenteric neuronal excitability. Data are shown as box-and-whisker plots (median, interquartile range, minimum–maximum). Significant differences between conditions, including comparisons between concentrations and buffer, are indicated (Dunn's multiple comparisons test; p-values shown). (**a**) Myenteric neurons: action potential number. Number of action potentials recorded in myenteric neurons following application of KBr at 100, 10, and 1 mg/mL and Krebs buffer solution. (**b**) Myenteric neurons: burst frequency (Hz). Burst frequency of myenteric neurons following application of KBr at 100, 10, and 1 mg/mL and Krebs buffer solution. (**c**) Myenteric neurons: duration of action potential firing (s). Duration of action

potential firing in myenteric neurons following application of KBr at 100, 10, and 1 mg/mL and Krebs buffer solution.

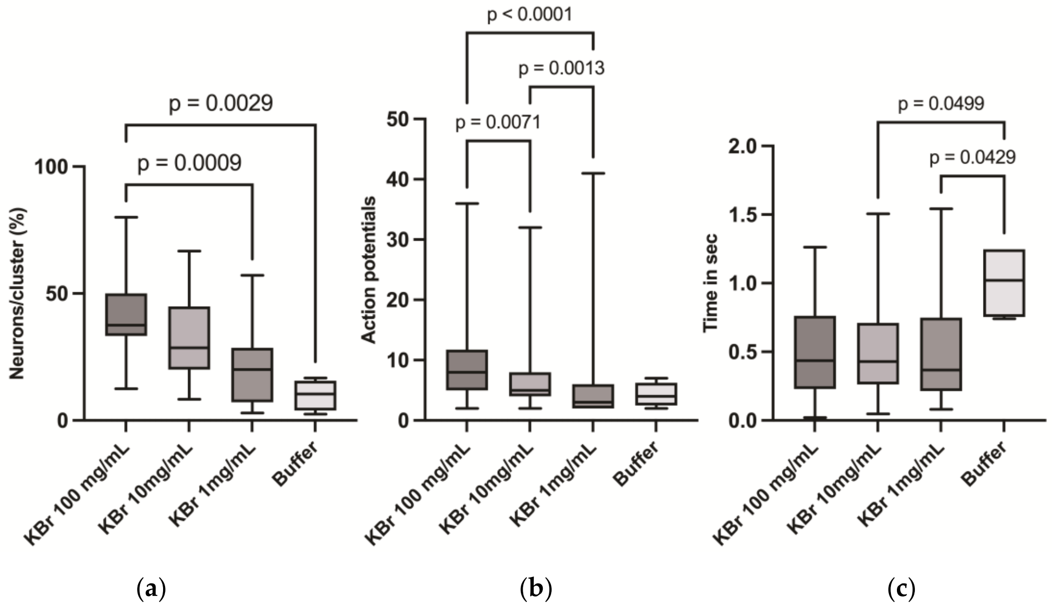

Figure S11. Effects of potassium bromide (KBr) on dorsal root ganglion (DRG) neuronal excitability. Data are shown as box-and-whisker plots (median, interquartile range, minimum–maximum). Significant differences between conditions, including comparisons between concentrations and buffer, are indicated (Dunn's multiple comparisons test; p-values shown). (**a**) DRG neurons: neurons per cluster (%). Percentage of DRG neurons per cluster exhibiting drug-induced changes in activity following application of KBr at 100, 10, and 1 mg/mL and Krebs buffer solution. (**b**) DRG neurons: action potential number. Number of action potentials recorded in DRG neurons following application of KBr at 100, 10, and 1 mg/mL and Krebs buffer solution. (**c**) DRG neurons: duration of action potential firing (s). Duration of action potential firing in DRG neurons following application of KBr at 100, 10, and 1 mg/mL and Krebs buffer solution.

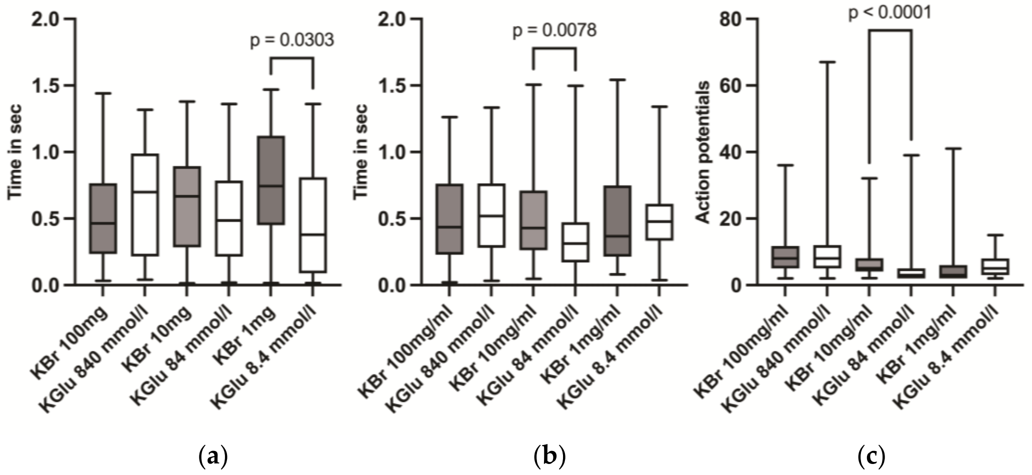

Figure S12. Comparison of drug-induced effects on neuronal excitability following application of potassium bromide (KBr) and potassium gluconate (KGlu) at equimolar potassium concentrations (100 mg/mL vs. 840 mmol/L, 10 mg/mL vs. 84 mmol/L, and 1 mg/mL vs. 8.4 mmol/L). Data are shown as box-and-whisker plots (median, interquartile range, minimum–maximum). Significant differences between conditions are indicated (Dunn's multiple comparisons test; p-values shown). (**a**) Myenteric neurons: duration of action potential firing (s). Duration of action potential firing in

myenteric neurons following application of KBr and equimolar KGlu. (**b**) DRG neurons: duration of action potential firing (s). Duration of action potential firing in dorsal root ganglion (DRG) neurons following application of KBr and equimolar KGlu. (**c**) DRG neurons: action potential number. Number of action potentials recorded in DRG neurons following application of KBr and equimolar KGlu.

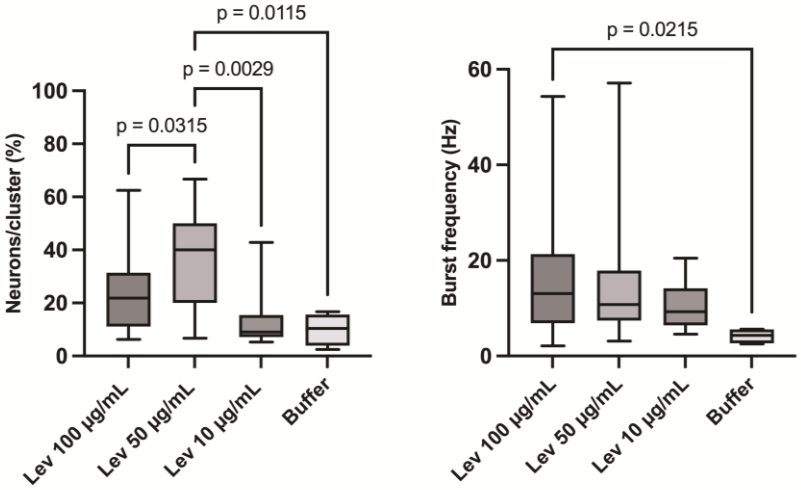

(**a**) (**b**)

Figure S13. Effects of Levetiracetam (Lev) on dorsal root ganglion (DRG) neuronal excitability. Data are shown as box-and-whisker plots (median, interquartile range, minimum–maximum). Significant differences between conditions are indicated (Tukey's test for panel a; Dunn's test for panel b; p-values shown). (**a**) DRG neurons: neurons per cluster (%). Percentage of DRG neurons per cluster exhibiting drug-induced changes in activity following application of Levetiracetam at 100, 50, and 10 µg/mL and Krebs buffer solution. (**b**) DRG neurons: burst frequency (Hz). Burst frequency of DRG neurons following application of Levetiracetam at 100, 50, and 10 µg/mL and Krebs buffer solution.

Table S1. Median change in action potential number (ΔAP number) after drug application relative to baseline activity.

**Compound**	**Cell type**	**Concentration level**	**Concentration**	**ΔAP number, median [IQR] (N)**
Phenobarbital	MS	Low	3.6 µg/mL	−2 [−3–+2] (N=32)
Phenobarbital	MS	Medium	16.8 µg/mL	−1 [−2–+3] (N=66)
Phenobarbital	MS	High	40 µg/mL	−1 [−2–+1.5] (N=31)
Phenobarbital	DRG	Low	3.6 µg/mL	+3.5 [+2–+7] (N=58)
Phenobarbital	DRG	Medium	16.8 µg/mL	+3 [+2–+6] (N=52)
Phenobarbital	DRG	High	40 µg/mL	+4 [+2–+8] (N=90)
KBr	MS	Low	1 mg/mL	+3 [+0.5–+7] (N=47)
KBr	MS	Medium	10 mg/mL	+4 [+2–+8] (N=83)
KBr	MS	High	100 mg/mL	+5 [+3–+8] (N=197)
KBr	DRG	Low	1 mg/mL	+2 [−2–+3.75] (N=54)
KBr	DRG	Medium	10 mg/mL	+5 [+4–+8] (N=138)
KBr	DRG	High	100 mg/mL	+7 [+4–+11] (N=94)
KGlu	MS	Low	8.4 mmol/L	0 [−2–+2.5] (N=71)
KGlu	MS	Medium	84 mmol/L	+4 [+3–+7] (N=136)
KGlu	MS	High	840 mmol/L	+7 [+3–+11] (N=90)
KGlu	DRG	Low	8.4 mmol/L	+4 [+2–+7.25] (N=96)
KGlu	DRG	Medium	84 mmol/L	+5 [+3–+9] (N=252)
KGlu	DRG	High	840 mmol/L	+8 [+5–+12] (N=252)

Imepitoin	MS	Low	10 µg/mL	−1 [−2–+2] (N=40)
Imepitoin	MS	Medium	50 µg/mL	−2 [−2.5–−0.25] (N=8)
Imepitoin	MS	High	100 µg/mL	−1 [−2–+2] (N=18)
Imepitoin	DRG	Low	10 µg/mL	+2 [+2–+3.75] (N=22)
Imepitoin	DRG	Medium	50 µg/mL	+2 [−2–+3] (N=12)
Imepitoin	DRG	High	100 µg/mL	−7 [−11–−5.5] (N=8)
Levetiracetam	MS	Low	10 µg/mL	−3 [−3.5–−1.5] (N=27)
Levetiracetam	MS	Medium	50 µg/mL	−2 [−2–−1] (N=29)
Levetiracetam	MS	High	100 µg/mL	−1 [−2–+1] (N=32)
Levetiracetam	DRG	Low	10 µg/mL	+2 [−2–+3] (N=26)
Levetiracetam	DRG	Medium	50 µg/mL	+5 [+3–+10] (N=73)
Levetiracetam	DRG	High	100 µg/mL	+5 [+2–+10] (N=76)
Topiramate	MS	Low	10 µg/mL	−2 [−3–−2] (N=35)
Topiramate	MS	Medium	50 µg/mL	−1 [−2–0] (N=33)
Topiramate	MS	High	150 µg/mL	−3 [−3.25–−2] (N=32)
Topiramate	DRG	Low	10 µg/mL	+2 [−2.25–+2] (N=12)
Topiramate	DRG	Medium	50 µg/mL	+2 [−3–+4] (N=23)
Topiramate	DRG	High	150 µg/mL	+2 [−4–+4] (N=13)
DMSO 1%	MS	Control	1%	−1 [−2–+0.5] (N=11)
DMSO 1%	DRG	Control	1%	+3 [+2–+4] (N=37)
Krebs buffer	MS	Control	single conc.	+1 [−1–+2] (N=13)
Krebs buffer	DRG	Control	single conc.	−2 [−5.5–+2.5] (N=11)

ΔAP number was calculated for each analyzed neuron as the number of APs after drug application minus the number of APs during baseline activity. Values are shown as median [IQR]. N indicates the number of analyzed neurons included in the paired before–after ΔAP number analysis for the respective condition. This N refers specifically to neurons analyzed for ΔAP number and is not identical to responder-cell, recorded-cluster, culture, dish, or animal numbers. Positive values indicate increased AP firing after drug application, whereas negative values indicate reduced AP firing.

Table S2. Sample structure for responder-cell, recorded-cluster, and independent-culture levels across experimental conditions.

Compound	Cell type	Concentration	Responder cells, n	Recorded clusters, n	Cell cultures, n
Phenobarbital	MS	3.6 µg/mL	12	37	3
Phenobarbital	MS	16.8 µg/mL	32	42	3
Phenobarbital	MS	40 µg/mL	11	48	4
Phenobarbital	DRG	3.6 µg/mL	51	41	3
Phenobarbital	DRG	16.8 µg/mL	47	49	3
Phenobarbital	DRG	40 µg/mL	79	67	3
KBr	MS	1 mg/mL	34	32	3
KBr	MS	10 mg/mL	72	32	3
KBr	MS	100 mg/mL	184	36	3
KBr	DRG	1 mg/mL	38	62	4
KBr	DRG	10 mg/mL	137	46	3
KBr	DRG	100 mg/mL	88	59	5
KGlu	MS	8.4 mmol/L	34	51	3
KGlu	MS	84 mmol/L	125	27	3
KGlu	MS	840 mmol/L	81	37	3
KGlu	DRG	8.4 mmol/L	84	50	3
KGlu	DRG	84 mmol/L	107	50	3
KGlu	DRG	840 mmol/L	238	49	4

Imepitoin	MS	10 µg/mL	10	49	4
Imepitoin	MS	50 µg/mL	2	28	3
Imepitoin	MS	100 µg/mL	6	27	4
Imepitoin	DRG	10 µg/mL	16	39	3
Imepitoin	DRG	50 µg/mL	7	40	3
Imepitoin	DRG	100 µg/mL	n.a.	36	3
Levetiracetam	MS	10 µg/mL	5	34	3
Levetiracetam	MS	50 µg/mL	5	33	3
Levetiracetam	MS	100 µg/mL	10	36	3
Levetiracetam	DRG	10 µg/mL	14	48	3
Levetiracetam	DRG	50 µg/mL	60	48	3
Levetiracetam	DRG	100 µg/mL	68	48	3
Topiramate	MS	10 µg/mL	1	34	3
Topiramate	MS	50 µg/mL	2	40	4
Topiramate	MS	150 µg/mL	n.a.	37	4
Topiramate	DRG	10 µg/mL	4	32	3
Topiramate	DRG	50 µg/mL	7	50	4
Topiramate	DRG	150 µg/mL	8	53	4
DMSO 1%	MS	1%	3	33	3
DMSO 1%	DRG	1%	29	46	3
Krebs buffer	MS	control	7	34	3
Krebs buffer	DRG	control	4	49	3

The table reports the available sample structure for each compound, concentration, and cell type. n indicates the count for the respective column: responder cells, recorded neuronal clusters, or independent primary cell cultures. These n values describe endpoint-specific sample structure and are not identical to the N of analyzed neurons reported in Supplementary Table S1 for ΔAP number analysis. Independent primary cell cultures represent the biological level of replication.

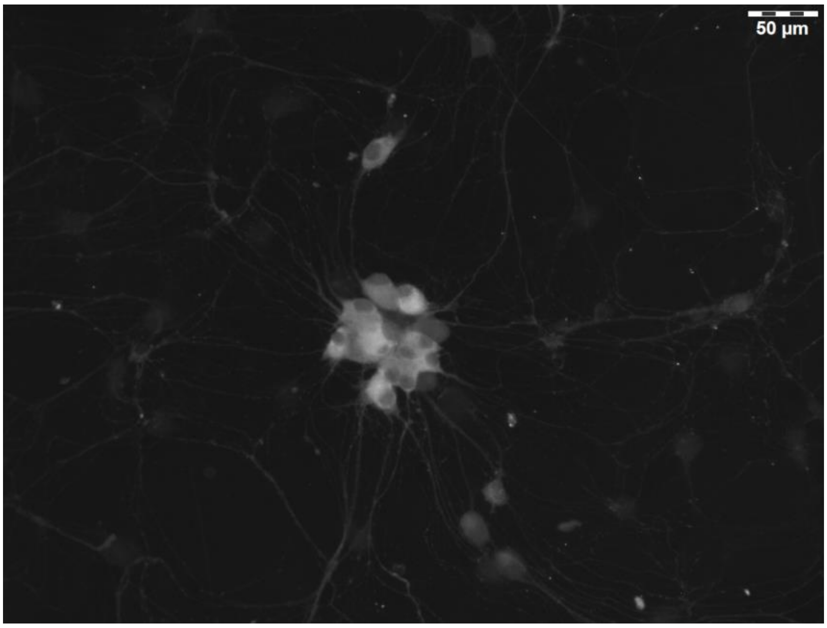

Figure S14. Representative NSE immunostaining of cultured myenteric neurons. Scale bar: 50 µm.

Table S3. Unsuccessful IHC cases and actively responding cells per experimental condition.

Compound	Cell type	Concentration	Actively responding cells, n	Unsuccessful IHC cells, n
Phenobarbital	MS	3.6 µg/mL	12	0

Phenobarbital	MS	16.8 µg/mL	32	1
Phenobarbital	MS	40 µg/mL	11	3
Phenobarbital	DRG	3.6 µg/mL	51	4
Phenobarbital	DRG	16.8 µg/mL	47	6
Phenobarbital	DRG	40 µg/mL	79	7
KBr	MS	1 mg/mL	34	1
KBr	MS	10 mg/mL	72	7
KBr	MS	100 mg/mL	184	7
KBr	DRG	1 mg/mL	38	8
KBr	DRG	10 mg/mL	137	10
KBr	DRG	100 mg/mL	88	34
KGlu	MS	8.4 mmol/L	34	2
KGlu	MS	84 mmol/L	125	4
KGlu	MS	840 mmol/L	81	7
KGlu	DRG	8.4 mmol/L	84	4
KGlu	DRG	84 mmol/L	107	6
KGlu	DRG	840 mmol/L	238	0
Imepitoin	MS	10 µg/mL	10	4
Imepitoin	MS	50 µg/mL	2	0
Imepitoin	MS	100 µg/mL	6	1
Imepitoin	DRG	10 µg/mL	16	0
Imepitoin	DRG	50 µg/mL	7	2
Imepitoin	DRG	100 µg/mL	0	1
Levetiracetam	MS	10 µg/mL	5	0
Levetiracetam	MS	50 µg/mL	5	3
Levetiracetam	MS	100 µg/mL	10	0
Levetiracetam	DRG	10 µg/mL	14	6
Levetiracetam	DRG	50 µg/mL	60	7
Levetiracetam	DRG	100 µg/mL	68	1
Topiramate	MS	10 µg/mL	1	2
Topiramate	MS	50 µg/mL	2	2
Topiramate	MS	150 µg/mL	0	1
Topiramate	DRG	10 µg/mL	4	4
Topiramate	DRG	50 µg/mL	7	4
Topiramate	DRG	150 µg/mL	8	2
DMSO 1%	MS	1%	3	0
DMSO 1%	DRG	1%	29	4
Krebs buffer	MS	control	7	0
Krebs buffer	DRG	control	4	2

The table summarizes the number of actively responding cells and cells with unsuccessful IHC staining for each compound, concentration, and cell type. Cells with unsuccessful IHC could not be included in IHC-linked phenotype or denominator analyses, but electrophysiological AP detection itself was independent of IHC staining.
